# Circulating extracellular vesicles as biomarkers in the diagnosis, prognosis and therapy of cardiovascular diseases

**DOI:** 10.3389/fcvm.2024.1425159

**Published:** 2024-09-02

**Authors:** Dominika Bernáth-Nagy, Melek Sükran Kalinyaprak, Evangelos Giannitsis, Pál Ábrahám, Florian Leuschner, Norbert Frey, Jona Benjamin Krohn

**Affiliations:** ^1^ Heart and Vascular Centre, Semmelweis University, Budapest, Hungary; ^2^ Department of Cardiology, Angiology and Pneumology, University Hospital Heidelberg, Heidelberg, Germany; ^3^ German Centre for Cardiovascular Research (DZHK) Partner Site Heidelberg/Mannheim, University of Heidelberg, Heidelberg, Germany

**Keywords:** extracellular vesicles, cardiovascular disease, diagnosis, prognosis, therapy, biomarker

## Abstract

Cardiovascular disease (CVD) ranks among the primary contributors to worldwide mortality. Hence, the importance of constant research on new circulating biomarkers for the improvement of early diagnosis and prognostication of different CVDs and the development and refinement of therapeutic measures is critical. Extracellular vesicles (EV) have a great potential as diagnostic and prognostic markers, as they represent their parent cell by enclosing cell-specific molecules, which can differ in quality and quantity based on cell state. Assuming that all cell types of the cardiovascular system are capable of releasing EV into circulation, an emerging body of evidence has investigated the potential role of serum- or plasma-derived EV in CVD. Comprehensive research has unveiled alterations in EV quantity and EV-bound cargo in the form of RNA, proteins and lipids in the context of common CVDs such as coronary artery disease, atrial fibrillation, heart failure or inflammatory heart diseases, highlighting their diagnostic and prognostic relevance. In numerous *in vitro* and *in vivo* models, EV also showed promising therapeutic potential. However, translation of EV studies to a preclinical or clinical setting has proven to be challenging. This review is intended to provide an overview of the most relevant studies in the field of serum or plasma-derived EV.

## 1 Introduction

Cardiovascular diseases (CVD) are one of the leading causes of death globally, accounting for almost a third of overall mortality (1). While advances in cardiovascular research in recent decades resulted in improved therapies for cardiovascular disease, boosting patient survival rates, a persistently high incidence of and overall mortality associated with CVD still warrants improved diagnostics, prognostication, and treatment of CVD. Cardiomyocytes are particularly sensitive to stress due their lack of renewal or reparative mechanisms, which poses the need for biomarkers capable of detecting subclinically affected myocardium to thus help with diagnosis and decision-making in the early stages of CVD. The development of novel prognostic biomarkers could contribute to the improvement of existing cardiovascular risk stratification, potentially leading to improved therapeutic or preventative treatment strategies.

Extracellular vesicles (EV), membrane bound non-replicable nanoparticles released by a multitude of different cell types representing their cell of origin, have moved into the spotlight as potential biomarkers or biomarker carriers in various cardiovascular disease entities. While EV is widely used as a generic term, numerous articles differentiate three main EV subgroups based on their subcellular origin. The smallest EV with a typical size range of 40–150 nm are termed exosomes (or also referred to as small EV or “sEV”), which are assumed to be of endosomal origin and thus derive from microvesicular bodies (2). Microvesicles (MV), ranging from 100 to 1,000 nm, are hypothesized to bud off directly from the plasma membrane and therefore express integrins and selectin on their surface (3). Having the largest size range of up to 5,000 nm, apoptotic bodies form directly from the plasma membrane during apoptosis (4). However, the Minimal Information for Studies of Extracellular vesicles (MISEV) of 2023 points out that assigning EV to a particular biogenesis pathway without any reliable imaging modality or specific separation method should be avoided (5). EV exert both auto- and paracrine effects, and their main function is assumed to mediate cell-cell and cell-matrix communication through their enclosed cargo encompassing RNA, lipids and proteins. EV content is altered depending on the state of the cell of origin as well as through environmental changes, and differentially enriched EV are involved in various pathophysiological processes such as immune responses, coagulation and atherosclerosis (6). A wide variety of EV isolation methods are described in literature, based on EV density, size or immunological characteristics (2). Commonly applied techniques are differential centrifugation (pelleting), as well as various biochemical precipitation methods (7). Recently, novel methods such as size-exclusion chromatography or immune-affinity isolation using specific capture antibodies have emerged as reliable methods for EV isolation (2). According to MISEV guidelines, numerous complementary methods should be used to quantitatively and qualitatively characterize isolated EV using the starting material as control (5). Recommended is the assessment of quantitative metrics of EV with e.g., light scattering methods like dynamic light scattering (DLS) or nanoparticle tracking analysis (NTA) with providing a limit of detection of the instrument, demonstration of EV-specific surface and intraluminal markers (e.g., CD9, CD63, CD81 or HSC70) and detection and reporting of co-isolated potential contaminant non-vesicular entities, such as lipoproteins (2, 5).

**Figure 1:**
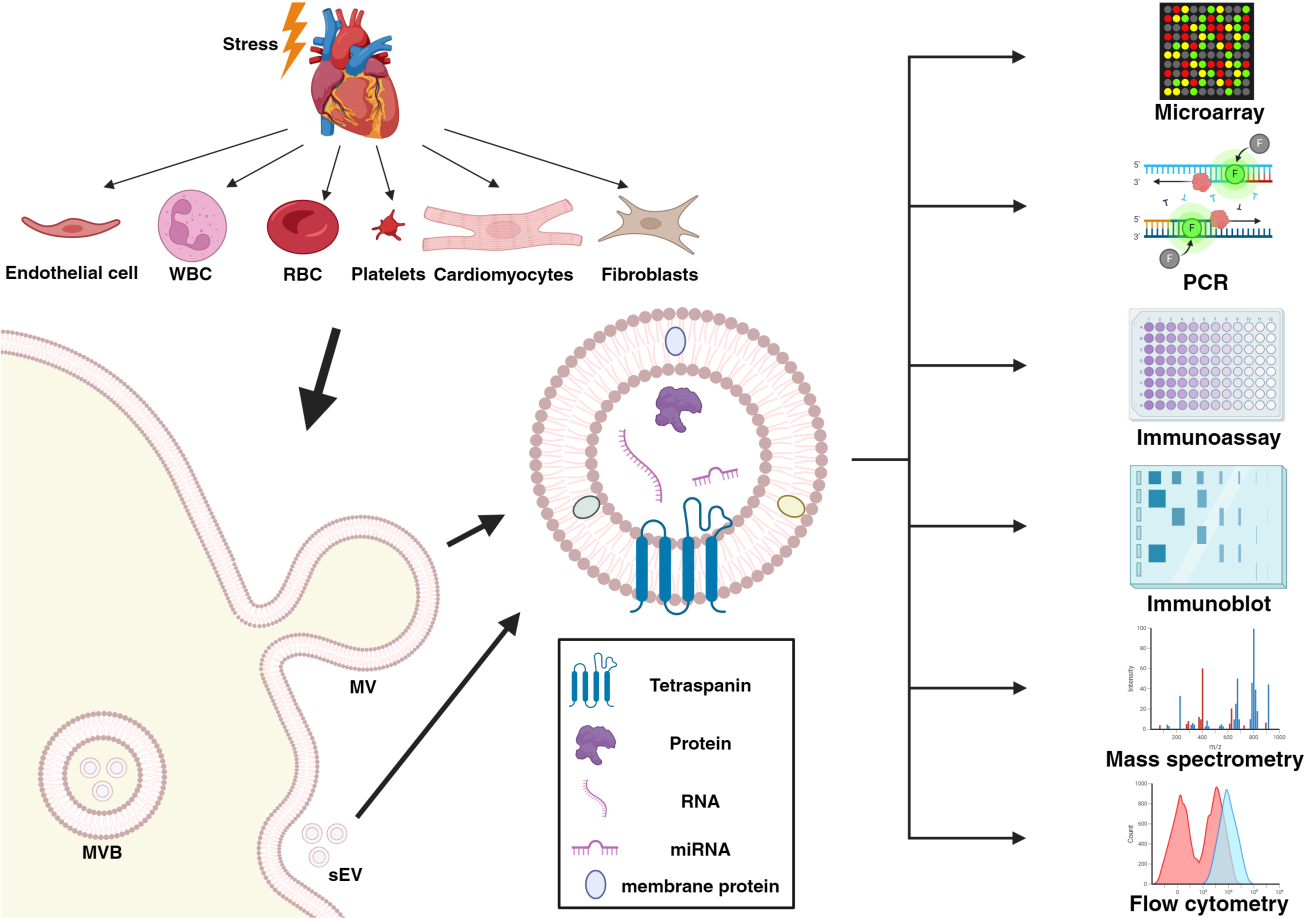
The biogenesis and exemplary analytical methods of extracellular vesicles released from all cell types of the cardiovascular system. Created with BioRender.com under publication license MS277LJMDV. WBC, white blood cell; RBC, red blood cell; MVB, multivesicular bodies; sEV, small extracellular vesicles; MV, microvesicles; RNA, ribonucleic acid; miRNA, micro-RNA; PCR, polymerase chain reaction.

All cell types of the cardiovascular system are assumed to be capable of releasing EV into circulation, and circulating EV reflect the cell and environmental state of the tissue of origin (7) (Figure 1). In recognition of their diagnostic potential, the number of studies analyzing EV quantity and cargo in the context of CVD is constantly growing. This review summarizes the latest and most relevant scientific articles about circulating EV in CVD, based on the potential usage of EV in the diagnostics and prognostics of CVD as well as exploring their monitoring potential following therapeutic measures.

## 2 Diagnostics

From purified EV isolates as a concentrated serum fragment, analysis of biomarker cargo is more convenient compared to using full serum or plasma, making EV a suitable biomarker source (2). Most studies have focused on EV-bound RNA and protein surface antigens, given the considerable amount of recent literature about these biomarkers in various disease entities. Due to their structural stability and subsequent longevity in circulation, RNA and especially micro-RNA have gained recent attention in the biomarker research field. EV-bound RNA is reportedly more stable and relatively more abundant than in free serum, encouraging the investigation of RNA compartmentalized in EV (8). EV are used as mediators of intercellular and cell-matrix communication, and a baseline EV release by cells under physiological circumstances was purported in previous literature (7). A change in the state of the cell of origin affects EV release not only quantitatively, but also in terms of specific EV cargo (9). The presented publications and relative abundance of the EV-associated markers in the diagnosis of CVD are summarized in Supplementary Table 2.

### 2.1 Coronary artery disease

Coronary artery disease (CAD) with its potentially fatal sequelae myocardial infarction and ischemic heart disease is among the most common causes of death worldwide, accounting for a majority of quality-adjusted life loss (10). The dynamic progression of CAD results in various clinical presentations categorized as acute (ACS) or chronic coronary syndrome (CCS), requiring different diagnostic tests and therapeutic strategies, respectively (11). In ACS, immediate decision making is crucial, as cardiomyocytes are particularly vulnerable to hypoxic stress, causing severe short and long-term effects cumulating in irreversible cell necrosis. Therefore, numerous clinical studies focus on the development and refinement of diagnostic tools for ACS, making circulatory EV a promising target in the search for novel diagnostic markers (7). Thus, this chapter aims to highlight recent studies advancing the concept of serum or plasma EV and EV-bound cargo as biomarkers in different stages of CAD. Selected publications and relative abundance of EV-associated protein and RNA markers in the diagnosis of CAD are summarized in Supplementary Tables 1A and 1B.

#### 2.1.1 EV protein and lipid cargo in the diagnosis of CAD

Zarà et al. (12) investigated size distribution and protein cargo of circulating plasma EV in patients with ST-segment elevation myocardial infarction (STEMI) compared to patients with baseline CCS. Nanoparticle tracking analysis revealed a significant increase in EV size and abundance in STEMI patients compared to patients with CCS. In addition, EV-bound glycoprotein IIb (GPIIb), VE-cadherin, ceruloplasmin and transthyretin were independently able to adequately discriminate between CCS and STEMI patients upon receiver operating characteristic analysis. In a more recent project, the authors demonstrated that plasma EV levels were elevated in plasma samples of patients with anterior STEMI, and circulating EV levels significantly correlated with the duration of myocardial ischemia (13). Gidlöf et al. analyzed the proteome of EV from the plasma of STEMI patients (*n* = 60) compared to healthy individuals (*n* = 22) (14). Using proximity extension assays, the authors identified three dysregulated EV-associated proteins, which were unaltered in the respective plasma: chymotrypsin C (CTRC), protooncogene tyrosine-protein kinase SRC (SRC) and C-C motif chemokine ligand 17 (CCL17). These findings were validated using plasma EV from CAD patients with varying disease severity, and EV-bound SRC was highlighted as a potential biomarker of advanced CAD. A different study found EV-bound connexin 43 (Cx43) to be differentially expressed in circulating EV of STEMI patients compared to healthy controls (15). Herein, lower Cx43 levels were found in EV isolated from blood samples of STEMI patients, while other EV-bound proteins remained unaltered between the two groups. As one of the most prominent ventricular gap junction proteins, the authors postulate that decrease in expression of EV-bound Cx43 results in adverse cell-cell communication in the heart in the setting of acute myocardial ischemia. Burrello et al. (16) demonstrated that EV sphingolipid composition successfully discriminates STEMI patients from healthy controls. Ceramide, sphingomyelin, and dihydroceramide levels were measured in the EV membrane using liquid chromatography tandem-mass-spectrometry (LC-MS/MS). All three sphingolipids were found abundant in the serum EV membrane of STEMI patients, significantly correlating with serum cardiac troponin T (hs-cTnT) levels, with a relative decrease following reperfusion. Cheow et al. (17) analyzed EV protein cargo of patients undergoing coronary artery bypass graft (CABG) surgery in the setting of either advanced CAD with stable angina pectoris (AP) or myocardial infarction using LC-MS/MS. The authors identified a total of six proteins involved in complement activation [complement C1q subcomponent subunit A (C1QA), complement C5], lipoprotein metabolism [apolipoprotein D (APOD) and C-III (APOCC3)] and platelet activation [platelet glycoprotein Ib alpha chain (GP1BA), platelet basic protein (PPBP)] to be significantly upregulated in serum EV of patients with myocardial infarction compared to patients with stable angina. He et al. (18) discovered significantly lower levels of low-density lipoprotein receptor (LDLR) and apolipoprotein A V (APOA5) in plasma-derived EV in samples of myocardial infarction patients compared to healthy controls. The authors verified their result in a cell culture experiment, where H9c2 cardiomyocytes showed blunted expression of LDLR and APOA5 protein following oxygen-glucose deprivation. Li et al. (19) investigated the potential of cysteine-rich protein 61 (Cyr61) in circulating EV in patients with ACS. Herein, the authors reported significantly higher EV-bound Cyr61 protein levels in serum samples of 210 ACS patients (including patients with unstable AP and myocardial infarction) compared to healthy controls. Additionally, they established a cell culture model of atherosclerosis utilizing human vascular smooth muscle cells (vSMC) treated with ox-LDL, where an siRNA-mediated knockdown of Cyr61 was performed to explore the effects of this protein *in vitro*. When exposed to ox-LDL, vSMCs showed enhanced cell viability, reduced apoptosis and increased migration, whereas knockdown of Cyr61 reversed all these effects, suggesting a regulatory role of Cyr61 in atherosclerosis. Dekker et al. (20) analyzed EV protein cargo of female patients presenting with CCS. EV were sequentially divided in three subfractions, with those co-precipitated with low-density lipoprotein (LDL) and high-density lipoprotein (HDL) being isolated separately. Additionally, EV was isolated from one subgroup devoid of LDL and HDL. Using immune bead assays, the authors measured the levels of the following EV-bound proteins in each subfraction: CD14, Cystatin C, Serpin C1, Serpin G1, Plasminogen, Serpin F2. Their results indicated that EV-bound CD14 and Cystatin C in both LDL and HDL subfractions and Serpin C1 and Serpin G1 in the HDL subfraction were significant predictors of stress-induced ischemia. The authors subsequently presented further data on EV cargo in the serum of patients presenting with acute chest pain and low serum hs-cTnI levels, where a significant inverse correlation between Cystatin C in plasma-derived EV and the incidence of myocardial ischemia was reported (21).

Numerous studies analyzed circulating non-coding RNAs as potential biomarkers in CAD (22–28), although in recent years the focus of RNA research increasingly shifted to the EV compartment, as RNA were reported to be more stable when enclosed in EV (8).

#### 2.1.2 EV-bound RNA in the diagnosis of CAD

##### 2.1.2.1 EV-bound micro-RNA cargo

Liu et al. (29) investigated serum EV-derived micro-RNA (miR) cargo of 62 patients with acute myocardial infarction (AMI; STEMI and Non-STEMI) alongside healthy controls. EV-bound miR-4516, miR-203 and their target transcript secretory frizzled-related protein 1 (SFRP1) levels were significantly higher in serum EV from the AMI group, with miR-4516 positively correlating with the SYNTAX score. Li et al. also screened differentially expressed miRs of circulating EV of ACS patients (STEMI, Non-STEMI and unstable AP) compared to healthy controls (30). In the EV from ACS patients, the authors found a significant increase in miR-146a expression while in the EV-depleted supernatant, this miR was barely detectable, hinting at a potential selective enrichment in the EV compartment. Wang et al. (31) demonstrated downregulation of miR-342-3p in plasma EV of AMI patients compared to healthy controls and thus purported a functional role in EV-mediated cardiomyocyte protection and repair by reducing apoptosis and autophagy rates. In search of a circulatory EV-bound miR with biomarker potential in samples of AMI and CCS patients, Su et al. (32) performed microarray screening in order to select target miRs, which they quantified with qRT-PCR. They found the levels of miR-1915-3p, miR-4507 and miR-3656 to be significantly decreased in AMI samples compared to CCS samples, and the expression of these miR showed good predictive accuracy. By analyzing the samples of AMI, stable AP and control patients, Zhao et al. (33) found that the levels of serum EV-bound miR-183 were significantly increased in both ischemic cardiovascular pathologies compared to control samples, showing the highest concentration in circulating EV of AMI patients. In search of a diagnostic biomarker for CCS, Han et al. (34) analyzed EV-bound miR from patients with CCS (*n* = 36) and matched healthy controls (*n* = 36). After small RNA sequencing of serum EV isolates and further validation of candidate miRs with qRT-PCR, the authors identified three significantly differentially expressed miR with ROC-AUC values >0.8 for the CCS group: let-7c-5p, miR-652-3p and miR-335-3p, the latter also positively correlating with the Gensini score, as a marker of CAD severity. Zhang et al. (35) investigated whether EV-bound miR involved in cardiovascular pathologies could have a diagnostic potential in CCS, and, analyzing plasma EV of CCS patients and patients in whom CCS was ruled out angiographically, the authors found three significantly differentially expressed miR: miR-942-5p, miR-149-5p and miR-32-5p. ROC analysis was performed to investigate the diagnostic value of these three miR for CCS prediction, and ROC-AUC of 0.693, 0.702 and 0.691 was found, respectively. Subsequent Gene Ontology Analysis, revealed involvement of the miR of interest in numerous biological pathways related to atherosclerosis, such as cell migration, angiogenesis and VEGFR signaling.

##### 2.1.2.2 Other (non-miR) RNA found in circulating EV

Besides miR, other types of RNA enclosed in EV have been investigated for their biomarker potential. He et al. (36) examined long RNAs in plasma EV from patients with AMI, CCS or healthy controls by sequencing analysis and identified two messenger RNAs that significantly distinguished the AMI and CCS groups from controls: ALPL and CXCR2, whose respective gene transcripts are involved in cardiac fibrosis and cardioprotection after ischemia-reperfusion injury. Further, the authors could demonstrate that EV-bound ALPL and CXCR2 mRNA levels showed substantial sensitivity in the prediction of AMI by ROC analysis, as well as significant correlation with baseline risk profile such as smoking history or blood neutrophil count, underlining their functional role in inflammatory processes. In a recent study, Wang et al. (37) screened differentially expressed circulating EV-bound circular RNAs (circRNAs) in the plasma of STEMI and control patients and found two circRNAs, exo-circ-0020877 and exo-circ-0009590, to be significantly upregulated in STEMI patients. Chen et al. (38) compared EV cargo of patients with STEMI, unstable AP and healthy controls using qRT-PCR and Western blot. In their study cohort, the authors demonstrated that serum EV long non-coding RNA (lncRNA) NEAT1 and matrix metalloproteinase 9 (MMP-9) levels were significantly higher in serum EV from STEMI patients compared to those from healthy controls, whereas EV-bound miR-204 levels negatively correlated with STEMI diagnosis. Zheng et al. (39), aiming to investigate the potential role of circulating EV-bound lncRNA in AMI, implemented sequencing profiles of serum EV from AMI patients and healthy controls and performed a two-fold validation on separate patient and control cohorts with qRT-PCR. Six lncRNA with the highest expressions were selected for validation, from which lncRNAs ENST00000556899.1 and ENST00000575985.1 were found to be significantly elevated in circulating plasma EV of AMI patients compared to healthy controls. Additionally, a more recent study from the same group included potential differentially expressed lncRNAs in serum EV isolates of CCS patients (40). This study encompassed a total of 218 participants with CCS and patients with non-cardiogenic chest pain (NCCP) as controls, following the same experimental protocol introduced in the previous study. Herein, the authors found lncRNAs ENST00000424615.2 and ENST00000560769.1 to be differentially expressed in serum EV from CCS patients compared to NCCP controls, the latter showing overexpression in EV samples from CCS patients with more progressed CAD. In the most recent study from this group, the authors investigated circulating EV-bound circRNA cargo of CCS patients alongside control patients diagnosed with NCCP, leading to the identification of two circRNAs significantly elevated in the EV of CCS patients: exo-hsa_circ_0075269, with a sensitivity and specificity for the diagnosis of CCS of 70% and 85%, respectively, and exo-hsa_circ_0000284, exhibiting a sensitivity and specificity for the diagnosis of CCS of 80% and 65%, respectively, highlighting the biomarker potential of EV-bound circRNA in the diagnosis of CCS (41).

### 2.2 Atrial fibrillation

Atrial fibrillation (AF) is the most common arrhythmia globally, with its prevalence increasing constantly in the last decades (42). In addition, it is associated with significant morbidity and mortality by causing an elevated risk for life-threatening complications such as ischemic stroke or heart failure (43–45). Diagnosis is made upon ECG documentation and the indication of the therapeutic measures is based on the evaluation of clinical presentation, clinical pattern of AF, underlying comorbidities and risk factors, and potential complications (43). Currently, there is a growing interest in biomarker research in the field of AF to expand current knowledge of disease pathophysiology. This endeavor holds potential for integrating novel discoveries into the diagnosis and risk evaluation of AF.

Wei et al. (46) revealed a notable upregulation of miR-92b-3p, miR-1306-5p and miR-let-7b-3p in serum-derived EV of AF patients, suggesting their potential involvement in the pathogenesis of AF. Despite a lack of experimental evidence to postulate a direct association of such miR with AF, PCR results indicated a significant increase in expression in the diseased group. Moreover, target genes associated with these miR were enriched in various related signaling pathways, such as Mitogen-Activated Protein Kinase (MAPK) and mammalian target of rapamycin (mTOR), suggesting a potential impact of these miR in AF development. The authors highlight the necessity for further research to further delve into the correlation between EV-derived miR and atrial fibrillation (AF), emphasizing its biomarker potential. Mun et al. (47) investigated differences in EV-derived miR in the serum of patients with persistent AF vs. control patients suffering from supraventricular tachycardia (SVT). Compared to SVT controls and patients with paroxysmal AF, patients with persistent AF exhibited a significant upregulation of 49 miR and downregulation of 4 miR in serum-derived EV. Of note, miR-107, -103a, -320d, -486, and let-7b demonstrated increased expression in patients with persistent AF, as confirmed by qRT-PCR. The above-mentioned miR are implicated in pathways that affect atrial function and structure, fibrosis and oxidative stress. MiR-107, -486 and let-7b were not standalone predictors of AF, with their potential functional link to AF pathogenesis related to structural changes in the atria. By contrast, miR-103a (adjusted OR: 2.29, 95% CI: 1.16–4.51, *P* = 0.02) and miR-320d (adjusted OR: 1.81, 95% CI: 1.23–2.67, *P* = 0.003) demonstrated independent associations with persistent AF, underscoring their autonomous roles in AF pathogenesis. Utilizing high-throughput sequencing, Wang et al. (48) identified 39 differentially expressed miR in serum EV from patients with AF compared to patients in sinus rhythm. Four of the differentially expressed and most abundant miR in cardiac tissue, namely miR-483-5p, miR-142-5p, miR-223-3p and miR-223-5p, were found to be differentially enriched in serum EV upon validation by RT-qPCR. While univariate logistic regression analysis revealed an association of miR-483-5p, miR-142-5p and miR-223-3p with AF, multivariate logistic regression implied that miR-483-5p independently correlates with AF prevalence. MiR-142-5p, miR-223-3p and miR-223-5p showed lower expression in serum EV of AF patients compared to patients in sinus rhythm, whereas a significant overexpression of miR-483-5p was found in EV of AF patients. Whilst a correlation between EV-bound miR concentration and the presence of AF could be appreciated, there was no statistical difference in total circulating EV between patients in AF and sinus rhythm. Ni et al. investigated the serum EV proteome in AF patients compared to healthy controls, identifying a total of 440 proteins expressed in serum EV. Using a fold change of ≥2.0 or ≤0.5 and a *p*-value <0.05 to denote statistical significance, the protein group significantly changing in abundance contained 39 elevated and 18 reduced proteins in AF, whereas a consistent presence/absence expression group encompassed 40 elevated and 75 reduced proteins. This study focused on understanding AF etiology, progression, and biomarkers at a molecular level, emphasizing altered pathways like coagulation, complement system and protein folding affected by the altered EV proteome. Protein disulfide isomerase (PDI), eukaryotic elongation factor 1 alpha (eEF1A) and prolyl cis-trans isomerase (PIN1) showed decreased expression in the serum EV of AF patients, indicating an imbalance in protein regulation, potential protein misfolding and oxidative stress, thus offering insights into AF pathogenesis (49). Chen et al. (50) uncovered increased levels of myocardial infarction-associated transcript (MIAT) expression within serum-derived EV from AF patients. MIAT binds to miR-485-5p, which in turn leads to the upregulation of C-X-C motif chemokine 10 (CXCL10). CXCL-10-overexpression promotes structural remodeling and consequently atrial fibrillation, therefore increased levels of MIAT in circulating EV might indicate a disease progression in AF patients.

### 2.3 Heart failure

Due to the increasing prevalence of cardiovascular risk factors such as obesity, old age, smoking, or diabetes mellitus, the prevalence of heart failure (HF) is constantly growing (51), unraveling a new need for specific biomarkers that are able to reliably predict HF. Alongside ongoing research on established plasma protein biomarkers such as brain natriuretic peptide (nt-proBNP) or cardiac troponin T (hs-cTnT), a growing number of studies focus on EV as promising potential biomarkers.

Zhang et al. (52) sought to find genetic biomarkers for the early diagnosis of dilated cardiomyopathy (DCM), as genetic mutations account for about 40% of all cases of DCM. Therefore, the authors analyzed EV-bound miR from the plasma of DCM patients with chronic heart failure (CHF) and healthy controls by miR sequencing and performed qRT-PCR for the top ten significantly dysregulated miR in an external validation cohort. Furthermore, through bioinformatic analyses, a functional role of the resulting miR of interest in various disease-related mechanisms such as oxytocin and hippo signaling pathways as well as in circadian regulation was purported. Wang et al. (53) examined fibroblast activity and viability through signaling pathways and miR in CHF. The authors showed that patients with CHF carry higher levels of EV-bound miR-320a, and co-culture of EV from HF patients or miR-320a overexpression in myocardial fibroblast cells demonstrated that miR-320a promotes fibroblast proliferation. A comprehensive study about plasma EV and their protein cargo in patients with cardiorenal syndrome was performed by Verbree-Willemsen et al. (54). The authors measured EV protein levels with immune assays from three EV subfractions and demonstrated that Serpin G1, Serpin F2, CD14 and Cystatin C were significantly associated with heart failure, the two latter biomarkers also correlating significantly with the combined incidence of renal and cardiac dysfunction. Wu et al. (55) focused on EV-bound miR as potential biomarkers of acute heart failure (AHF) in DCM patients by analyzing serum samples of 43 DCM patients alongside healthy controls. Serum EV-bound miR-92b-5p levels were increased in DCM-AHF patients, correlating with patient age and several echocardiographic parameters such as left ventricular (LV) systolic and diastolic diameter, left atrial diameter, LV ejection fraction and LV fractional shortening. In another study, the authors hypothesized that EV-bound miR-92b-5p could also serve as biomarker for HFrEF patients hospitalized with AHF (56). Roura et al. (57) investigated the proteomic signature of serum EV in DCM patients using LC-MS/MS and demonstrated that fibrinogen, serotransferrin, alfa-1-antitrypsin and apolipoproteins were more abundant in serum EV of DCM patients compared to healthy controls.

### 2.4 Myocarditis

The most severe and acute form of myocarditis is fulminant myocarditis (FM). Due to its rapid onset and potential serious complications (such as HF and potentially fatal cardiogenic shock), immediate diagnostic and therapeutic measures are crucial. The gold standard diagnostic method to date is endomyocardial biopsy (EMB), which is time and resource consuming, and also very invasive with a relatively high risk of adverse events (58–60). Additional, readily available diagnostic tools such as cardiac specific proteins (hs-cTnT, nt-proBNP), inflammatory markers, ECG, or echocardiography are unspecific for the diagnosis of myocarditis (60), thus vindicating a constant demand for reliable non-invasive biomarkers.

Zhang et al. (58) investigated the role of serum EV-bound miR in myocarditis and their potential as biomarkers in the samples of pediatric patients with fulminant myocarditis (FM), clinically diagnosed in accordance with current guidelines. They identified that hsa-miR-146a-5p, hsa-miR-23a-3p and hsa-miR-27a-3p, which reportedly play a regulatory role in the interaction between immune cells and cardiomyocytes, were overexpressed in serum EV from FM patients. In a study with more than 100 adult patients with FM, EV-bound hsa-miR-155 and hsa-miR-320a were also shown to be significantly differentially expressed in FM patients compared to healthy controls (61). Interestingly, EV-bound hsa-miR-320a was also shown to be elevated in samples of CHF patients and, as described previously, postulated to play a significant role in fibrosis (53).

### 2.5 Cardiac allograft rejection

Cardiac allograft rejection (CAR), despite the immunosuppressive therapy and monitoring, remains one of the most serious complications in the early post-transplant period in heart transplant (HTX) patients (62). For decades, regular endomyocardial biopsy (EMB) has been the gold-standard procedure for monitoring CAR, however, two non-invasive blood-based methods, gene expression profiling (GEP) and donor-derived cell-free DNA (dd-cfDNA) detection have emerged as additional diagnostic entities to reduce the need for invasive and complication-prone EMB (63, 64). Additionally, comprehensive research has been carried out in the EV field to investigate the diagnostic potential of these circulating particles in monitoring acute cellular (ACR) and antibody mediated rejection (AMR).

Celik et al. (65) investigated novel diagnostic targets in ACR after HTX by analyzing the EV cytokine cargo of HTX patients with and without ACR. The authors found EV-bound tumor necrosis factor-related weak inducer of apoptosis (TWEAK) to be elevated in serum EV of patients with incident ACR, underlining the role of TWEAK in the activation of proinflammatory genes and cytokine release. Castellani et al. (66) focused on serum EV surface markers measured with multiplex flow cytometry to develop a novel methodology in the diagnosis of acute allograft rejection in HTX patients. The authors identified 11 differentially expressed EV surface antigens in acute rejection and control cases, and by using a machine learning algorithm, established a model that demonstrated excellent diagnostic performance in allocating patients with cellular and antibody mediated rejection and HTX patients without allograft rejection. Kennel et al. (67) demonstrated that allograft rejection could alter serum EV protein expression patterns in HTX patients, discovering several differentially expressed proteins by LC-MS/MS predominantly associated with immunological and hematological pathways including complement activation and coagulation.

## 3 Prognostication

In the relentless exploration of potential biomarkers for prognostication of CVD, EV have increasingly shifted into the spotlight of contemporary research. The following passages aim to provide an overview of the latest and most relevant studies about EV as potential prognostic biomarkers in various cardiovascular pathologies, including CAD, AF, LV remodeling, HF and major adverse cardiac events. Selected studies and relative abundance of EV-associated markers in the prognostication of CVD are summarized in Supplementary Table 3.

### 3.1 Coronary artery disease

Management of coronary artery disease (CAD) requires comprehensive prognostic assessments to guide therapeutic interventions observing associated risks and benefits to thus improve overall patient outcomes. As a result of the increasingly sensitive diagnostics and fast therapeutic measures, a positive trend towards higher survival rates of CAD and its sequelae is observed, thus simultaneously challenging Western healthcare systems with a growing cohort of CAD patients at risk of substantial morbidity and mortality (1, 68). Therefore, diagnostic tools for adequate prognostication and risk stratification of patients with CAD is in high demand. Recent work has investigated EV as a potential circulatory biomarker in this context.

Systemic inflammatory response syndrome (SIRS) is postulated to be a substantial contributing factor to the high mortality observed in patients treated with venoarterial extracorporeal membrane oxygenation (VA-ECMO) due to circulatory failure. Siegel et al. (69) investigated the prognostic value of circulating EV released by the cell populations that contribute to SIRS in patients treated with VA-ECMO and STEMI patients following reperfusion. Using flow cytometry, the authors demonstrated that elevated total Annexin V^+^ EV on day 1 of VA-ECMO treatment is predictive of mortality, and increased cardiomyocyte-derived caveolin-3^+^ EV count on the first day after reperfusion in STEMI patients was associated with impaired left ventricular function as negative prognostic indicator. Zara et al. (13) intended to find prognostic markers for patients following revascularization in STEMI patients. Herein, the authors found smaller sEV size and lower expression of platelet derived markers CD41-CD61 to correlate with reperfusion injury assessed by cardiac magnetic resonance (CMR), associated with a diminished prognosis related to functional recovery. Han et al. (70) aimed to investigate the differentially expressed circulating EV-bound miR cargo in samples of CAD patients with hyperglycemia and found hsa-let-7b-5p to be a significant discriminator of normo- and hyperglycemic CAD patients, thus implying prognostic relevance with regard to CAD severity correlating with patient-specific SYNTAX scores. Zheng et al. (40) not only showcased the diagnostic potential of circulating EV-bound lncRNAs described earlier, in the case of lncRNA ENST00000560769.1, a prognostic potential was postulated, as this EV-bound lncRNA was significantly elevated in CCS patients with more progressed disease, correlating with established risk stratification scores suggestive of a limited prognosis.

### 3.2 Major adverse cardiac events

The term “major adverse cardiac events” (MACE) by non-universal definition includes life-threatening CVD entities such as non-fatal or fatal MI, any non-fatal or fatal ischemic or hemorrhagic stroke or any cardiovascular death from other causes (hemodynamically significant arrhythmias, fatal cardiogenic shock, fatal abdominal aortic aneurysm rupture, sudden cardiac death etc.) (71, 72). Patients with established CAD have a significantly higher risk of developing MACE with an incidence ranging from 21% to 49% (73). Risk factors for MACE undergo ongoing investigation in patients with CAD (72, 74, 75) and those undergoing cardiac (76, 77) or non-cardiac (72, 78–81) surgery. Recently, EV as biomarkers or biomarker carriers have been investigated with respect to MACE in various patient cohorts.

Jansen et al. (82) investigated EV-bound miR cargo of CCS patients and demonstrated that the levels of EV-bound miR-126 and miR-199a were inverse predictors of MACE during a six-year follow-up period, as increased levels of these EV-bound miR were associated with a reduced risk of MACE. In addition to the excellent diagnostic efficacy described previously, Wang et al. (37) demonstrated that STEMI patients with elevated EV-bound circ-0020887 and EV-bound circ-0009590 levels exhibited a lower MACE-free 1-year survival rate, as these two circ-RNAs were postulated to be independent risk factors for adverse cardiovascular outcomes. Timmerman et al. (71, 83) sought to improve existing risk stratification for post-operative MACE in patients undergoing carotid endarterectomy (CEA) by analyzing differences in protein cargo of EV from pre-operative plasma samples. Higher levels of CD14, Cystatin C, Serpin F2 and Serpin C1 in HDL-co-precipitated-EV were associated with increased risk of MACE in a three-year follow-up period, additional inclusion of existing CV risk factors significantly improved the predictive value of EV-bound CD14 levels with regard to incident MACE post CEA (71). The authors also quantified ceramide and phosphatidylcholine (PC) content of the EV membrane and found that distinct ceramide/PC ratios measured preoperatively could predict postoperative risk for MACE in a three-year follow-up period (83). These results could be evaluated for optimized secondary preventative therapeutic measures in affected patients. In a recent study from the same research group, protein expression in plasma, circulating EV and atherosclerotic plaque tissue samples of patients undergoing CEA underwent comparative analysis (84). EV contained the most differentially expressed proteins (*n* = 21) correlated to incident MACE in a 3-year follow-up period. Oggero et al. (85) investigated the prognostic potential of EV for MACE in a hypertensive patient cohort treated with atorvastatin or placebo. Herein, higher CD14^+^ and CD14^+^CD41^+^EV levels were found to be associated with an increased risk of MACE, which conversely was found to be attenuated in patients on statin therapy. Through specific EV markers, the authors hypothesize monocyte- and platelet-mediated cross-talk to exert an adverse effect on CVD risk in hypertensive patients. Recent data by Suades et al. (86) suggests that activated proinflammatory and prothrombotic state reflected by circulating leukocyte- and platelet-derived MV in patients with familial hypercholesterolemia (FH) prognosticates cardiovascular events (CVE) and overall mortality. Quantitative analysis of MV populations phenotyped by cell of origin from liquid biopsies of 150 patients with or without incident CVE demonstrated that the total number of Annexin V^+^MV, white blood cell type-derived MV (CD15^+^), activated platelet-derived MV (PAC1^+^, CD62P^+^, TSP1^+^) and pan-leukocyte-derived MV (CD45^+^) are increased, whereas activated monocyte-derived MV (CD14^+^/11b^+^) were significantly reduced in the circulation of FH patients developing CVE over a follow-up period of 3.3 ± 2.6 years. Escate et al. (87) reported a role for EV-derived miR-133a in the prognosis of CVE in patients with familial hypercholesterolemia (FH) over a follow-up period of 8 years. Analysis of blood samples from more than 300 FH patients and non-FH matched controls revealed elevated levels of EV-miR-133a in patients suffering CVE (AUC = 0.76, HR = 3.89). Further in silico and *in vitro* analyses suggested that miR-133a-mediated effects may be functionally associated with gene expression regulation of cell-membrane lipid-receptor LRP6 and inflammatory cytokines, such as CXCL8, IL6 and TNF.

### 3.3 Atrial fibrillation

Current ESC guidelines recommend the management of AF patients based on the integrated ABC pathway, where “C” stands for the assessment of cardiovascular risk factors and concomitant diseases (43). The following studies on EV research in AF suggest that serum EV and EV-bound cargo could hold a valuable role in early evaluation of comorbidities and pathological states such as thrombogenicity or nephrotoxicity, therefore assisting physicians in the prognostic assessment of AF.

Mork et al. (88) demonstrated that EV, specifically those containing tissue factor (TF), display procoagulant characteristics. The median level of TF-bearing EVs were significantly higher in the plasma of AF patients compared to non-AF control samples. Additionally, AF patients demonstrated an approximate 40% increase in median plasma TF concentration and a 30% rise in median plasma von Willebrand factor (vWF) concentration. These results imply a potential mechanistic association between circulatory EV and heightened thrombogenicity in AF patients. The abundance of TF-carrying EV poses an elevated risk of thrombosis, providing a potential predictive biomarker for AF-associated thromboembolism. Siwaponanan et al. (89) identified elevated levels of EV in the 101–200 nm size range in AF patients compared to non-AF controls. Large EV miR profiling analysis demonstrated 19 significantly upregulated and 21 significantly downregulated EV-bound miR in AF patients. Six highly expressed miR, i.e., miR-339-3p, miR-106b-3p, miR-378a-3p, miR-590-5p, miR-328-3p and miR-532-3p, were validated, and logistic regression analysis demonstrated increased miR levels to be linked with AF and increased arrhythmogenesis through progressive tissue remodeling. The increased levels of large EV might furthermore contribute to a prothrombic state in AF. Lau et al. (90) found elevated levels of endothelial/platelet-derived EV in the sera of AF patients on anticoagulation to be closely associated with worsening renal function. This increase in endothelial-derived EV demonstrated a gradual escalation across stages 1–4/5 of chronic kidney disease, subsequent to a significant negative correlation with estimated glomerular filtration rate (eGFR). Notably, endothelial-derived EV emerged as the only independent predictor of renal function, unlike the other investigated markers of a prothrombotic state and cellular activation, including platelet-derived EV, soluble *P*-selectin, and soluble E-selectin levels, which showed no significant differences across varying levels of renal dysfunction in AF patients. The findings of this study underscore a possible link between endothelial function, indicated by circulating endothelial-derived EV levels, and renal function in patients with AF, making them a potential indicator of renal disease in AF.

### 3.4 Heart failure

Due to advances in diagnosis and therapy of cardiovascular disease in recent decades, patients with ACS tend to have higher survival rates, leading to an increased prevalence of post-MI HF due to adverse cardiac remodeling (68, 91). Cardiac remodeling post-MI is characterized by morphological changes in structure and shape, subsequent to impairment of cardiac function (92, 93). In all patients with ACS, a comprehensive echocardiographic examination is routinely recommended to evaluate ventricular and valvular function as well as wall motion abnormalities, and several plasma proteins, such as hs-cTnT/TnI and NT-proBNP are measured for prognostic assessment (94). In addition, current investigations focus on novel biomarker candidates functionally involved in the pathophysiological processes causing adverse cardiac remodeling, thus serving as potential diagnostic or prognostic targets.

Lim et al. investigated (95) whether LDL-co-precipitated EV carry prognostic markers for LV remodeling preceding HF after AMI. The authors analyzed coagulation and fibrinolysis pathway-related proteins in circulating plasma LDL-EV by immunoassay and found that 1 month after MI, EV-bound von Willebrand factor (vWF):plasminogen and SerpinC1:plasminogen ratios hold better prognostic power for reverse LV remodeling than routine serum biomarkers NT-proBNP, hs-cTnT or hs-cTnI. Pursuing the same objective, Gasecka et al. (96) reported decreased concentrations of circulating EV from endothelial cells, red blood cells and platelets identified by established surface markers CD146, CD235a and CD61, respectively in plasma samples of patients with MI who developed adverse LV remodeling within 6 months. Based on these results, the authors postulate that the reduced number of circulating EV may serve as a prognostic factor for cardiac homeostasis perturbation. Zheng et al. (39) performed sequencing profiles for differentially expressed EV-bound lncRNAs in MI and control patients and, following validation with qRT-PCR, the authors found that lncRNA ENST00000575985.1 could serve as a prognostic biomarker for the development of severe HF after MI. Wang et al. (97) investigated cardiac fibrosis as one of the main contributors to adverse cardiac remodeling in HF. The authors found EV-bound miR-425 and miR-744 to be significantly downregulated in plasma-derived EV of HF patients, and in an *in vitro* model of cardiac fibroblasts stimulated with angiotensin II to induce fibrosis, decreased expression levels of the aforementioned two miR was observed. Consequently, inhibition of these miR caused an increase of pro-fibrotic gene and protein expression patterns, while overexpression of miR-425 and miR-744 was able to repress this upregulation. Based on these results, the authors concluded that suppressed levels of the aforementioned EV-bound miR may predict cardiac fibrosis as a hallmark of adverse cardiac remodeling post MI. Dang et al. (98) examined the effect of remote ischemic conditioning (RIC) after MI on angiogenesis by utilizing a rat model of MI with or without reperfusion complementary to analysis of blood samples of patients with MI undergoing PCI or without reperfusion. Randomly selected rats and patients from previously mentioned cohorts underwent RIC by limb ischemia. By analyzing circulating EV, EV-bound lncRNA TUG1 was found to be upregulated after MI, and to have a significant role in the suppression of angiogenesis by downregulating the HIF-1α/VEGF-α axis. By applying RIC to affected rats or patients, the authors demonstrated lower EV-bound TUG1 levels, leading to enhanced endothelial function and angiogenesis after MI. Thus, EV-bound TUG1 could not only pose a prognostic marker of disease severity after MI, but also open novel therapeutic approaches to improve angiogenesis. In accordance with previous studies (99, 100), Suades et al. (101) showed that not all populations of circulatory EV present surface phosphatidylserine (PS) due to lack of externalization during EV formation. The authors further explored dichotomy of PS^+^ and PS^−^ circulating EV from different parental cells in the context of chronic HF (cHF). Blood-derived EV from 119 cHF patients alongside 21 non-HF control patients were analyzed by flow cytometry. cHF patients displayed increased levels of total PS^−^ EV, endothelial CD31^+^/PS^−^ EV, leukocyte-derived CD45^+^/PS^−^ EV, lymphocyte-derived CD3^+^/PS^−^ EV, neutrophil-derived CD15^+^/PS^−^ EV, natural killer cell-derived CD56^+^/PS^−^ EV, reflecting an elevated inflammatory state characteristic of cHF (102), and markers of tissue and cell damage, as well as interstitial gap junction connexin-43 CX43^+^/PS^−^ EV compared to controls. Interestingly, CD31^+^/PS^+^ EV was proven to be decreased in cHF, thereby suggesting the possibility to be considered an independent biomarker population functionally different from CD31^+^/PS^−^ EV. A more recent paper from the same group (103) investigated differential PS exposure on platelet-derived circulating EV in the context of a chronic (chronic HF) and acute (ACS) cardiovascular disease. CD31^+^/PS^+^ EV and CD41^+^/PS^+^ EV were found to be increased in cHF patients compared to non-HF controls upon quantitative analysis, and total PS^−^ EV and CD31^+^/PS^+^ EV to be increased in cHF compared to ACS, whereas total PS^+^ EV were increased in ACS patients. Based on their findings about the EV-PS content, the authors are postulate that increased PS^+^ EV levels could reflect a prothrombogenic state commonly found in the setting of ACS, FH or ischemic stroke, whereas higher relative concentration of PS^−^ EV is found more commonly in nonatherosclerotic diseases. These findings in platelet-derived circulatory EV may bear implications in the diagnosis and prognosis of acute and chronic CVD.

## 4 Therapeutics

Numerous studies demonstrated the promising cardioprotective effect of stem cell- or cardiac progenitor cell-derived EV following ischemic/hypoxic injury in various *in vitro* and *in vivo* models (104–110). We herein intend to introduce recent findings on potential therapeutic effects of serum- or plasma-derived EV. This overview therefore includes studies exploring the potential of circulatory EV in prognosis or therapeutic monitoring (e.g., following drug administration) by quantitative analysis of circulating EV or characterization of EV-specific cargo.

### 4.1 The effects of circulating EV *in vitro* and *in vivo*

D'Ascenzo et al. (111) established a model to examine the cardioprotective effect of human serum-derived EV on ischemia reperfusion injury (IRI) by eliciting remote ischemic preconditioning (RIPC) in ACS patients. The RIPC protocol consisted of manual blood pressure cuff inflation cycles on 15 individuals from a population of 30 ACS patients, while the other half of the cohort went through a sham procedure of minimal cuff inflation. After the procedure, blood was drawn, serum EV were collected and used in an *in vitro* hypoxia-reoxygenation model of H9c2 rat myoblasts and in *ex-vivo* isolated rat hearts following IRI. After coculture with EV isolates for 2 h, cell viability and infarct size were found to be increased when cocultured with RIPC-treated serum EV, while reduced after incubation with sham-treated EV. The authors thus demonstrate that human serum-derived EV from ACS patients bear the potential to modulate tissue response to IRI both *in vitro* and *ex vivo*. Lombardo et al. (112) examined the potential therapeutic use of endothelial-derived EV in enhancing angiogenesis. In their *in vitro* study, IL-3 positively regulated endothelial cell-derived EV release, and these EV facilitated paracrine pro-angiogenic signals leading to endothelial tube formation through miR-126-3p, pre-miR-126 and activated signal transduction and activator of transcription 5 (pSTAT5). Li et al. (113) investigated potential cardioprotective and anti-apoptotic effects of circulating EV isolated from the plasma of AMI patients (MI-exo) and healthy controls (Con-exo) co-incubated with hypoxic H9c2 cells and primary mouse cardiomyocytes or injected intramyocardially into AMI mice. In the *in vitro* study, the authors focused particularly on ferroptosis of cardiomyocytes, a distinct, iron-dependent regulated form of cell death that has been introduced as an important pathway in the progression of ischemic heart disease. Compared to Con-exo, MI-exo significantly attenuated hypoxia-induced ferroptosis in cardiomyocytes *in vitro*. In the murine AMI model, intracardial injection of MI-exo was found to improve cardiac function and reduce infarct size following AMI. Measured with qRT-PCR, miR-26b-5p expression was significantly downregulated in MI-exo and overexpression of this miR in cardiomyocytes *in vitro* resulted in silencing the effect of MI-exos. Targetscan, dual luciferase reporter gene assay and miR-26b-5p overexpression revealed solute carrier family 7 member 11 (SLC7A11) to be the direct target of this miR. SLC7A11 is a member of biological pathways involved in cystine uptake, gluthatione synthesis and ferroptosis resistance, propagating such pathways as potential therapeutic targets in ischemic heart disease. Gu et al. (114) proposed a novel therapeutic target for AMI through serum EV-bound miR-21. Serum EV were isolated from healthy donors and added to cultured rat cardiomyocytes exposed to oxygen-glucose deprivation and subsequent reperfusion (OGD/R model). Co-culture with EV isolates was found to attenuate cardiomyocyte apoptosis following OGD/R. Enrichment of miR-21 in human serum EV and treatment of cardiomyocytes with miR-21 inhibitors *in vivo* confirmed the previously described antiapoptotic effect of this miR. Li et al. (115) sought to investigate the effect of serum EV of patients with myocardial ischemia on angiogenesis *in vitro* as well as in a murine limb ischemia model. Patients were recruited after diagnostic coronary angiography and enrolled in the ischemic (>70% stenosis) or in the control group (<50% or no stenosis) and EV (isc-Exo and con-Exo) were isolated from an intracoronary blood draw. Human umbilical vein endothelial cells (HUVEC) were cultured and co-incubated with serum-derived EV. In the *in vitro* study, isc-Exo significantly bolstered endothelial cell proliferation, migration and tube formation compared to con-Exo, indicating that serum EV from patients with myocardial ischemia might aide in the distribution of pro-angiogenic mediators. In a mouse model of unilateral hind limb ischemia, human serum EV were injected into the muscle of the affected limb, which subsequently underwent histological analysis. Isc-Exo significantly enhanced neovascularization and promoted blood flow. Microarray analysis showed significantly lower miR-939-5p levels in isc-Exos, and through transfection of miR-939-5p inhibitor and mimetic, the authors showcased a crucial regulatory effect of this miR in angiogenesis by regulating the nitric oxide signaling pathway. Beyond the diagnostic potential of EV, Parent et al. (116) demonstrated that EV administration could be a promising therapeutic approach to prevent the onset of atrial fibrillation. One of the most common complications following open-chest surgery is new-onset postoperative atrial fibrillation (POAF) attributed to inflammation in the pericardium. The authors observed that in mice with sterile pericarditis induced by talc administration, trans-epicardial injection of human EV into atrial tissue reduced the likelihood of AF onset by 40% 3 days post-surgery. The cargo within these EV was found to be enriched with 83 miRs and 196 proteins associated with reduced inflammation and fibrosis. EV treatment led to a decrease in the recruitment of CD11b^+^ neutrophils, CD3^+^ cytotoxic T cells and CD4^+^ T helper cells. Simultaneously, EV administration promoted macrophage polarization towards pro-reparative anti-inflammatory CD163^+^ “M2” macrophages, while leading to a decrease in pro-inflammatory “M1” macrophages.

### 4.2 Circulating EV as biomarkers in therapeutic response and prognosis

#### 4.2.1 The potential role of circulating EV during pharmacotherapy in CVD

Weiss et al (117). investigated the anti-inflammatory effect of factor Xa inhibitor rivaroxaban by investigating circulating plasma sEV in a population of patients with nonvalvular atrial fibrillation treated with either rivaroxaban or warfarin (controls). NTA showed that concentration of circulating sEV was reduced in plasma of patients treated with rivaroxaban. Label-free proteomic profiling of plasma EV from rivaroxaban-treated patients revealed a decrease in pro-inflammatory proteins, e.g., of the S100 calcium binding protein family, and a reciprocal increase in negative regulators of inflammation such as complement 4 binding protein, compared to patients treated with warfarin. Additionally, lower levels of soluble *P*-selectin were measured in the sera of rivaroxaban-treated patients, which alongside its anti-inflammatory effect derived from EV proteomic profiling, postulates an inhibitory role of rivaroxaban on endothelial cell activation. Liani et al. (118) examined the potential effect of chronic low-dose aspirin treatment on circulating EV release within a 24-h interval on a population of patients with high cardiovascular risk. The authors reported a transient reduction in circulating serum EV count measured with flow cytometry 10 h after aspirin gavage, which was followed by a rapid recovery after 24 h. These findings validated the result of Bulut et al. (119), which purported a significant reduction in circulating platelet- and endothelial cell-derived EV under chronic aspirin treatment, hypothesizing that this mechanism may contribute to vascular repair processes of endothelial progenitor cells. The effect of statin treatment on circulating EV was assessed by Suades et al. (120). Using flow cytometry, the authors found significantly lower levels of EV expressing markers of various cell origins, such as platelet, endothelial cell and leukocyte. Through their findings, the authors postulate that statin treatment may be conducive to an attenuated vascular cell activation via an EV-dependent mechanism.

Depending on individual thromboembolic risk, patients with atrial fibrillation may require life-long treatment with anticoagulants. Several research groups explored the impact of various medications on EV and their role in the pathogenesis of atrial fibrillation. Chirinos and colleagues (121) found elevated levels of endothelial-derived EV in patients with non-valvular atrial fibrillation under antiarrhythmic therapy with digoxin compared to no digoxin use, indicating increased endothelial cell activation under cardiac glycoside therapy as a potential harbinger for vascular thrombosis. Based on the study by Lenart-Migdalska et al. (122), dabigatran administration is associated with an increase in platelet-derived microparticles (PMPs) positive for CD42b, thus suggesting increased platelet activation. The same research group observed that individuals receiving rivaroxaban for non-valvular atrial fibrillation showed an increase in PMP and endothelial-derived MP (EMP) levels. Interestingly, statin therapy seemed to have a positive effect, potentially attenuating the release of PMPs and EMPs induced by rivaroxaban, thus potentially diminishing its thrombogenic potential (123). These investigations propose that circulating EV may serve as a promising prognostic biomarker anticipating pharmaceutical treatment response in patients, thus optimizing therapeutic monitoring.

#### 4.2.2 Circulating EV as biomarker in interventional and operative therapeutic measures

Cardiomyocyte-derived circulating CD172a^+^ EV in calcific aortic valve stenosis patients provides additional prognostic information after transcatheter aortic valve replacement (TAVR) (124). In a cohort of 312 patients scheduled for TAVR, Anselmo et al. demonstrated that elevated preprocedural serum CD172a^+^ EV count, even in patients with elevated hs-cTnT and NT-proBNP levels, is associated with a favorable prognosis in the first postprocedural year. In the comprehensive study by Siegel et al. (69) described earlier, EV profile was analyzed on the first day of VA-ECMO therapy in patients with circulatory failure, and elevated total serum Annexin V^+^ EV levels were found to be associated with a less favorable prognosis. The previously introduced studies by Timmermann et al. (71, 83) focused on the potential prognostic markers carried by circulating plasma EV related to postoperative MACE following CEA. Elevated levels of CD14, Cystatin C, Serpin F2 and Serpin C1 in HDL-co-precipitated-EV (71) and distinct ceramide/phosphatidylcholine ratios measured in the membrane fraction of plasma EV were linked to an elevated MACE risk within 3 years post-OP (83). Femmino et al. (125) demonstrated that PCI could alter circulating EV cargo in patients with unstable angina or NSTEMI undergoing remote ischemic pre-conditioning (RIPC), thus affecting cardio-protective pathways. Plasma EV of patients, who did not receive RIPC before PCI showed reduced levels of the cardioprotective transcript DUSP6, in addition, circulating EV of patients who underwent RIPC before reperfusion had increased expression of genes involved in stress responses and attenuation of cell cycle progression. These findings may hint at a negative effect of PCI on the postulated ability of circulating EV to induce cardioprotection in the setting of ischemia.

Shichao Li et al. (126) showcased that EV-mediated crosstalk between myofibroblasts and cardiomyocytes enhanced vulnerability to atrial fibrillation by ionic remodeling mediated by EV-bound miR-21-3p. In accordance with former study, Yao et al. (127) illustrated a cardioprotective effect upon inhibition of EV release through sphingomyelinase inhibitor GW4869 in a canine model of AF. Herein, 20 specimen were assigned to three different groups: sham, pacing and pacing + intravenous GW4869 administration. The pacing and pacing + GW4869 groups were exposed to atrial pacing with a frequency of 450 beats/min for 7 days. In both groups, atrial pacing increased EV release. Elevated EV-derived miR-21-5p concentrations were linked to lower expression of tissue inhibitor of metalloproteinase 3 (TIMP3) and increased expression of transforming growth factor-ß1 (TGF-ß1), thus contributing to structural remodeling. Inhibition of EV secretion and thus reduction of EV-bound miR-21-5p cargo was found to alleviate atrial fibrosis. Siwaponanan et al. (89) hypothesized that enrichment of miR-378a-3p, miR-590-5p and miR-106b-3p in circulating EV may elevate the risk of AF by promoting structural cardiac remodeling, specifically fibrosis, hypertrophy and associated dysrhythmia. The other three validated miRs in the study, miR-328-3p, miR-532-3p and miR-339-3p, were proposed to be associated with large EV secretion upon cardiomyocyte activation or apoptosis.

## 5 Conclusion

This review aims to provide a comprehensive overview of the latest studies on circulating serum- or plasma-derived EV in the context of cardiovascular disease with a special focus on diagnostic, prognostic and therapeutic implications. In the last decade, circulating EV were introduced as a novel biomarker and biomarker carrier possibly eluding current serum diagnostics. EV encompass a wide variety of molecular cargo that might be of both diagnostic and prognostic significance, such as proteins involved in various functional pathways, coding and non-coding RNAs as well as lipids. This cargo serves an essential role in cell-cell communication, offering a mode of delivery of information about the state of their cell of origin. As all cell types of the cardiovascular system are assumed to be capable of releasing EV, the interest in exploring the quantitative and qualitative changes in cardiac tissue-derived EV in circulation during initiation and progression of cardiovascular disease is regarded as a promising asset in future diagnostics. Herein, we report on numerous studies investigating coronary artery disease, atrial fibrillation, heart failure, myocarditis and cardiac allograft rejection with regard to the diagnostic and prognostic potential of circulating EV and their cargo.

An important aspect to consider in the interpretation of EV-based studies is the method of EV isolation, which has been shown to exert a significant impact on quantitative EV yield and EV cargo (128). Choosing the most suitable EV isolation method highly depends on the biological starting material and its quantitative and qualitative characteristics (5). For instance, while ultracentrifugation is deemed more suitable for voluminous samples, there is a higher risk of EV damage, affecting quantitative analysis, whereas precipitation-based methods may result in higher EV yield at the cost of increased co-isolation of contaminants (7). Therefore, the MISEV recommends a full preanalytical detail with regard to EV isolation method to ensure adequate interpretation of results (5). To foster consistency and transparency in the field of EV research, field-specific databases such as EVpedia (129) or Vesiclepedia (130) have been introduced, and authors are urged to upload their findings (131). These attempts at standardizing the field may curb limitations resulting from preanalytical variance.

One further aspect to consider in the interpretation and comparative analysis of circulatory EV research are the preanalytical conditions of the initial blood draw. There is a significant difference in quantitative and qualitative EV yield from serum and plasma samples, as without supplementation of an anticoagulant, platelets release an additional fraction of EV upon activation (132), revealing a significant fraction of platelet-derived EV in serum that is not present in plasma. This is conducive to a relative increase of platelet-derived EV-cargo proteins, RNA and lipids in bulk analyses of serum samples, inevitably affecting serum EV fraction profiles (133, 134). For studies investigating platelet-derived EV and their cargo, serum poses a suitable choice as starting material, as markers associated with platelet activation are reportedly more abundant in serum (135). For studies involving the qualitative analysis and particularly quantification of non-platelet derived EV, EV isolation from plasma samples seems more ideal, as the population of activated platelet-derived EV is diminished through avoidance of serum sample preparation (132). Another point to contemplate in this respect is the anticoagulant of choice for plasma samples. While EDTA and citrate are most widely used in EV research, these calcium chelators promote association of EV with platelets, thus lowering absolute EV yield from plasma samples (136). For the aforementioned reasons, it is highly recommended to consider the starting material of choice based on the objective of subsequent analyses.

Little information is available to date on the applicability of blood-derived EV as a therapeutic tool in the treatment of CVD. However, the results provided herein promulgating the cardioprotective potential of circulatory EV both *in vitro* and *in vivo* indicate that this field warrants further exploration and evaluation for future therapeutic use.
