# Supplementary material for: Circulating extracellular vesicles as biomarkers in the diagnosis, prognosis and therapy of cardiovascular diseases
Source: Front Cardiovasc Med. 2024 Sep 2;11:1425159. doi: 10.3389/fcvm.2024.1425159 (PMC11417624; doi:10.3389/fcvm.2024.1425159)
Supplement: Supplementary file 1 [file Datasheet1.docx]

Supplementary Material

# Supplementary Figures and Tables

**Table 1a** Overview of the relative abundance of EV protein and lipid cargo of potential diagnostic relevance in different subset of CAD patients

EV: extracellular vesicle, sEV: small EV, STEMI: ST-elevation myocardial infarction, CCS: chronic coronary syndrome, NTA: nanoparticle tracking analysis, AUC: area under curve, CI: confidence interval, GPIIb: glycoprotein IIb, CTRC: chymotrypsin C, SRC: protooncogene tyrosine-protein kinase SRC, CCL17: C-C motif chemokine ligand 17, ns: non-significant, WB: western blot, IP: immunoprecipitation, ELISA: enzyme-linked immunosorbent assay, Cx43: connexin 43, LC-MS/MS: liquid chromatography tandem mass spectrometry, CABG: coronary artery bypass graft surgery, MI: myocardial infarction, AP: angina pectoris, C1QA: complement C1q subcomponent subunit A, APOD: apolipoprotein D, APOCC3: apolipoprotein C-3, GP1BA: platelet glycoprotein Ib alpha chain, PPBP: platelet basic protein, LDLR: low-density lipoprotein receptor, APOA5: apolipoprotein A V, Cyr61: cysteine-rich protein 61, OR: odds ratio, LDL: low-density lipoprotein, HDL: high-density lipoprotein, TEX: tiny extracellular fraction, uAP: unstable AP, ECLIA: electrochemiluminescence immunoassay

| Reference | Groups [N] | EV subpopulation | Detection method [unit of measurement] | EV-bound target | Study finding (relative abundance) |
| --- | --- | --- | --- | --- | --- |
| Zara et al. 2021 (12) | STEMI [35]; CCS [32] | plasma sEV | immuno-blotting [A.U./ml; Arbitrary Unit] | GPIIb | STEMI 210 (97.36-298.3), CCS 0 (0-77.41) p<0.0001 |
|  |  |  |  | VE-cadherin | STEMI 243.3 (177.3-522.6), CCS 103.1 (44.84-156.1) p<0.0001 |
|  |  |  |  | ceruloplasmin | STEMI 459.8 (342.1-679.3), CCS 663.8 (474.4-950.2) p=0.016 |
|  |  |  |  | transthyretin | STEMI 461 (274.6-884.7), CCS 1613 (912.7-2459) p=0.0004 |
|  |  |  |  | fibronectin | STEMI 240.6 (171.3-324.7), CCS 326.2 (236.4-412.8) p=0.036 |
| Gidlöf et al. 2019 (14) | discovery cohort: STEMI [60]; healthy controls [22]; validation: stable AP [8]; uAP [8]; STEMI [8] | plasma EV | proximity extension assay; mean relative abundance | CTRC | STEMI 2.13, control 2.634 p=0.0006 |
|  |  |  |  | SRC | STEMI 0.531, control 1.04 p=0.0006 |
|  |  |  |  | CCL17 | STEMI 1.513, control 2.441 p=0.001 |
| Martins-Marques et al. 2020 (15) | STEMI [28]; healthy control [29] | serum and plasma EV | WB-IP, ELISA | Cx43 | decreased in STEMI p=0.0051 |
| Burrello et al. 2020 (16) | STEMI [7]; healthy control [9] | plasma sEV | LC-MS/MS [pmol/mL] | ceramide | STEMI 110.1 (97.4-179.9), control 39.7 (32.8-59.1) p<0.05 |
|  |  |  |  | dihydroceramide | STEMI 5.0 (3.7-6.9), control 1.8 (1.4-2.4), p<0.05 |
|  |  |  |  | sphingomyelin | STEMI 621.0 (570.2-934.9), control 191.6 (162.9-261.2), p<0.05 |
| Cheow et al. 2016  (17) | validation cohort: patients undergoing CABG with MI [15] or stable AP [20]; validation: MI [17]; stable AP [26] | plasma EV | LC-MS/MS; validation: antibody-based assays [pg/mL] | C1QA | MI 2.2E^7^ ± 1.246E^7^, control 1.189E^7^ ± 6.256E^6^, p=0.005 |
|  |  |  |  | complement C5 | MI 1.702E^6^ ± 1.154E^6^, control 6.994E^4^ ± 8.498E^4^, p=0.0047 |
|  |  |  |  | APOD | MI 1.468E^7^ ± 7.328E^6^, control 9.572E^6^ ± 6.572E^6^, p=0.0267 |
|  |  |  |  | APOCC3 | MI 3.224E^9^ ± 7.266E^8^, control 2.431E^9^ ± 1.083E^9^, p=0.0064 |
|  |  |  |  | GP1BA | MI 3.852E^7^ ± 1.079E^7^, control 2.819E^7^ ± 9.653E^6^, p=0.0031 |
|  |  |  |  | PPBP | MI 4.002E^6^ ± 2.737E^6^, control 2.455E^6^ ± 1.626E^6^, p=0.0465 |
| He et al. 2022 (18) | MI [30]; healthy controls [30] | plasma sEV | ELISA | LDLR | lower in MI p<0.001 |
|  |  |  |  | APOA5 | lower in MI p<0.001 |
| Li et al. 2021 (19) | ACS (uAP 160; MI 50); healthy controls [50] | plasma sEV | WB, ELISA | Cyr61 | elevated in ACS (AUC=0.763 95% CI=0.705-0.822 p<0.01) |
| Dekker et al. 2020 (20) | CCS [187]; healthy controls [257] | LDL plasma subfraction sEV | immuno-bead assay [pg/mL] | CD14 | CCS 22888 [18223-29183], control 21558 [17208-27288], (OR 1.54, 95% CI=1.09-2.16 p=0.014) |
|  |  |  |  | Cystatin C | CCS 15178 [12498-18131], control 13909 [11638-16432], (OR 1.70, 95% CI=1.17-2.46 p=0.006) |
|  |  | HDL plasma subfraction sEV |  | CD14 | CCS 6515 [5116-8221], control 6394 [5257-8023], (OR 1.46, 95% CI=1.02-2.11 p=0.042) |
|  |  |  |  | Cystatin C | CCS 1802 [1312-2779], control 1653 [1199-2699], (OR 1.35, 95% CI=1.04-1.76 p=0.026) |
|  |  |  |  | Serpin C1 | CCS 1615100 [1026650-2880050], 1599700 [874794-2436300], (OR 1.31, 95% CI=1.04-1.66 p=0.024) |
|  |  |  |  | Serpin G1 | CCS 1837500 [1363750-2454850], 1698300 [1250400-2506500], (OR 1.37, 95% CI=1.04-1.80 p=0.027) |
| Dekker et al. 2020 (21) | uAP [83]; healthy controls [186] | TEX plasma subfraction sEV | ECLIA | Cystatin C | uAP 22.89 (4.70), control 23.98 (4.93) (square root transformation of values),  (OR 0.93 95% CI=0.88-0.99) |

**Table 1b** Overview of the relative abundance of EV RNA cargo of potential diagnostic relevance in different subset of CAD patients

mi-RNA-seq: micro-RNA sequencing, RT-qPCR: real time quantitative PCR, miR: micro-RNA, SFRP1: secretory frizzled-related protein 1, CAD: coronary artery disease, MMP-9: matrix metalloproteinase 9

| Reference | Groups [N] | EV subpopulation | Detection method [unit of measurement] | EV-bound target | Study finding (relative abundance) |
| --- | --- | --- | --- | --- | --- |
| Liu et al. 2023 (29) | validation cohort: MI [62]; healthy controls [31] | plasma sEV | miRNA-seq; validation: RT-qPCR | miR-4516 | elevated in MI AUC=0.9809 p<0.0001 |
|  |  |  |  | miR-203 | elevated in MI AUC=0.6823 p=0.006 |
|  |  |  | ELISA | SFRP1 | elevated in MI AUC=0.9603 p<0.0001 |
| Li et al. 2021 (30) | ACS (STEMI, NSTEMI, uAP 21; 23; 19); healthy controls [25] | plasma sEV | RT-qPCR | miR-146a | elevated in ACS STEMI (AUC=0.8305 95% CI=0.6853-0.9756 p=0.0001) NSTEMI (AUC=0.8696 95% CI=0.7659-0.9732 p<0.0001) uAP (AUC=0.7916 95% CI= 0.6506-0.9326 p=0.001) |
| Wang et al. 2021  (31) | validation cohort: MI [12]; healthy controls [9] | plasma sEV | miRNA-seq; validation: RT-qPCR | miR-342-3p | decreased in MI p<0.05 |
| Su et al. 2020 (32) | validation cohort: MI [30]; CCS [30] | plasma sEV | microarray; validation: RT-qPCR relative expression level | miR-4507 | MI 0.622 (0.162-1.011), CCS 0.909 (0.679-1.317) p=0.02 |
|  |  |  |  | miR-1915-3p | MI 0.530 (0.366-0.966), CCS 1.001 (0.789-1.116) p<0.01 |
|  |  |  |  | miR-3656 | MI 0.697 (0.378-0.957), CCS 1.021 (0.943-1.108 p)<0.01 |
| Zhao et al. 2019 (33) | validation cohort: MI [14]; stable AP [10]; healthy controls [14] | plasma sEV | microarray; validation: RT-qPCR | miR-183 | decreased in controls, compared to diseased patients p<0.05 ; elevated in MI compared to stable AP p<0.01 |
| Han et al. 2023 (34) | validation cohort: CCS [36]; healthy controls [36] | plasma sEV | miRNA-seq; validation: RT-qPCR | let-7c-5p | decreased in CCS (AUC=0.8472 95% CI=0.7534-0.9411 p<0.0001) |
|  |  |  |  | miR-335-3p | elevated in CCS (AUC=0.8029 95% CI=0.7014-0.9043 p<0.0001) |
|  |  |  |  | miR-652-3p | decreased in CCS (AUC=0.8009 95% CI=0.7005-0.9013 p<0.0001) |
| Zhang et al. 2020 (35) | CCS [20]; healthy controls [20] | serum sEV | RT-qPCR relative abundance | miR-942-5p | CCS −5.6 ± 0.8, control −6.2 ± 0.9, p=0.038 |
|  |  |  |  | miR-149-5p | CCS −6.1 ± 0.7, control −6.7 ± 0.7, p=0.021 |
|  |  |  |  | miR-32-5p | CCS −5.4 ± 0.9, control −6.1 ± 0.9, p=0.032 |
| He et al. 2021 (36) | MI [10]; CAD [8]; healthy controls [10] | plasma sEV | RNA sequencing | ALPL | elevated in MI (AUC=0.99 95% CI=0.9484-1.000 p=0.0002) |
|  |  |  |  | CXCR2 | elevated in MI (AUC=0.98 95% CI=0.8478-1.000 p=0.0005) |
| Wang et al. 2023 (37) | STEMI [64], healthy controls [64] | plasma sEV | microarray and miRNA-seq datasets screened; validation: RT-qPCR | exo-circ-0020887 | elevated in STEMI (AUC=0.85 p<0.001) |
|  |  |  |  | exo-circ-0009590 | elevated in STEMI (AUC=0.80 p<0.001) |
| Chen et al. 2020 (38) | STEMI [47]; uAP [24], healthy controls [27] | serum sEV | RT-qPCR expression fold change | NEAT1 | expression fold change STEMI 2.95 ± 2.47, control 1.00 ± 0.75, p<0.01, uAP 1.05 ± 0.85, p<0.01 |
|  |  |  |  | miR-204 | expression fold change STEMI 0.64 ± 0.40, control 1.00 ± 0.56, p<0.01, uAP 0.64 ± 0.4, p<0.05 |
|  |  |  | immunoblotting expression fold change | MMP-9 | expression fold change STEMI 3.58 ± 3.17, control 1.00 ± 0.38, p<0.01, uAP 1.71 ± 0.87, p<0.05 |
| Zheng et al. 2020 (39) | 2. validation cohort: MI [85]; healthy controls [48] | plasma sEV | RNA-seq; validation: RT-qPCR fold change | ENST00000556899.1 | upregulated in MI (p=0.04, fold change=2.49) |
|  |  |  |  | ENST00000575985.1 | upregulated in MI (p=0.008, fold change=3.14) |
| Zheng et al. 2023 (40) | 2. validation cohort: CCS [100]; controls [48] | plasma sEV | RNA-seq; validation: RT-qPCR fold change | ENST00000424615.2 | upregulated in CCS (p=0.063, fold change=2.39) |
|  |  |  |  | ENST00000560769.1 | upregulated in CCS (p=0.003, fold change=3.07) |
| Liu et al. 2023 (41) | 2. validation cohort: CCS [100]; controls [48] | plasma sEV | RNA-seq; validation: RT-qPCR relative abundance | exo-hsa_circ_0075269 | upregulated in CCS 14.78 (7.96-38.06), control (n.a.) p<0.01 |
|  |  |  |  | exo-hsa_circ_0000284 | upregulated in CCS 2.52 (1.14-7.53), control (n.a.) p<0.01 |

**Table 2** Overview of selected publications about the diagnostic potential of EV in CVD.

EV: extracellular vesicle, sEV: small EV, STEMI: ST-elevation myocardial infarction, CCS: chronic coronary syndrome, NTA: nanoparticle tracking analysis, AUC: area under curve, CI: confidence interval, GPIIb: glycoprotein IIb, CTRC: chymotrypsin C, SRC: protooncogene tyrosine-protein kinase SRC, CCL17: C-C motif chemokine ligand 17, ns: non-significant, WB: western blot, IP: immunoprecipitation, ELISA: enzyme-linked immunosorbent assay, Cx43: connexin 43, LC-MS/MS: liquid chromatography tandem mass spectrometry, CABG: coronary artery bypass graft surgery, MI: myocardial infarction, AP: angina pectoris, C1QA: complement C1q subcomponent subunit A, APOD: apolipoprotein D, APOCC3: apolipoprotein C-3, GP1BA: platelet glycoprotein Ib alpha chain, PPBP: platelet basic protein, LDLR: low-density lipoprotein receptor, APOA5: apolipoprotein A V, Cyr61: cysteine-rich protein 61, OR: odds ratio, LDL: low-density lipoprotein, HDL: high-density lipoprotein, TEX: tiny extracellular fraction, uAP: unstable AP, ECLIA: electrochemiluminescence immunoassay, mi-RNA-seq: micro-RNA sequencing, RT-qPCR: real time quantitative PCR, miR: micro-RNA, SFRP1: secretory frizzled-related protein 1, CAD: coronary artery disease, MMP-9: matrix metalloproteinase 9, AF: atrial fibrillation, SR: sinus rhythm, SVT: supraventricular tachycardia, MIAT: myocardial infarction-associated transcript, DCM: dilated cardiomyopathy, HF: heart failure, CHF: chronic heart failure, GEO: National Center for Biotechnology Information Gene Expression Omnibus, HMDD: Human MicroRNA Disease Database, sST2: soluble growth stimulation expressed gene 2, AHF: acute heart failure, HFrEF: heart failure with reduced ejection fraction, FM: fulminant myocarditis, ACR: acute cellular rejection, HTX: heart transplantation, TWEAK: tumor necrosis factor-related weak inducer of apoptosis, AMR: antibody-mediated rejection, HLA: human leukocyte antigen

| Reference | Groups [N] | EV subpopulation | Detection method | EV-bound target or variable | Study finding (statistical significance) |
| --- | --- | --- | --- | --- | --- |
| Zara et al. 2021 (12) | STEMI [35]; CCS [32] | plasma sEV | NTA | EV dimensions (mode) | elevated in STEMI (AUC=0.850 95% CI=0.75-0.95 p<0.0001) |
|  |  |  | immuno-blotting | GPIIb | elevated in STEMI (AUC=0.896 95% CI=0.82-0.97 p<0.0001) |
|  |  |  |  | VE-cadherin | elevated in STEMI (AUC=0.876 95% CI=0.78-0.98 p<0.0001) |
|  |  |  |  | ceruloplasmin | elevated in CCS (AUC=0.836 95% CI=0.72-0.96 p<0.0001) |
|  |  |  |  | transthyretin | elevated in CCS (AUC=0.841 95% CI=0.73-0.96 p<0.0001) |
| Zara et al. 2023 (13) | STEMI [42] from which anterior STEMI [19] | plasma sEV | NTA | EV concentration | elevated in anterior STEMI (p=0.0001 (t-test)) and in late revascularization (p=0.038 (t-test)) |
| Gidlöf et al. 2019 (14) | discovery cohort: STEMI [60]; healthy controls [22]; validation: stable AP [8]; uAP [8]; STEMI [8] | plasma EV | proximity extension assay; validation: ELISA | CTRC | elevated in controls (AUC=0.76 95% CI=0.265-0.862 p=0.014); validation: ns |
|  |  |  |  | SRC | elevated in controls (AUC=0.74 95% CI=0.328-0.888 p=0.015); validation: decreased in STEMI compared to AP (p<0.05 (ANOVA)) |
|  |  |  |  | CCL17 | elevated in controls (AUC=0.73 95% CI=0.169-0.660 p=0.002); no validation performed |
| Martins-Marques et al. 2020 (15) | STEMI [28]; healthy control [29] | serum and plasma EV | WB-IP, ELISA | Cx43 | decreased in STEMI (p=0.0051 (Mann-Whitney test)) |
| Burrello et al. 2020 (16) | STEMI [7]; healthy control [9] | plasma sEV | LC-MS/MS | ceramide | elevated in STEMI (AUC=0.968 95% CI=0.890-1.000) |
|  |  |  |  | dihydroceramide | elevated in STEMI (AUC=0.921 95% CI=0.765-1.000) |
|  |  |  |  | sphingomyelin | elevated in STEMI (AUC=0.968 95% CI=0.890-1.000) |
| Cheow et al. 2016  (17) | validation cohort: patients undergoing CABG with MI [15] or stable AP [20]; validation: MI [17]; stable AP [26] | plasma EV | LC-MS/MS; validation: antibody-based assays | C1QA | upregulated in MI (p=0.012; validation: p=0.005 (t-test)) |
|  |  |  |  | complement C5 | upregulated in MI (p=0.087; validation: p=0.0047 (t-test)) |
|  |  |  |  | APOD | upregulated in MI (p=0.033; validation: p=0.0267 (t-test)) |
|  |  |  |  | APOCC3 | upregulated in MI (p=0.029; validation: p=0.0064 (t-test)) |
|  |  |  |  | GP1BA | upregulated in MI (p<0.0001; validation: p=0.0031 (t-test)) |
|  |  |  |  | PPBP | upregulated in MI (p=0.027; validation: p=0.0465 (t-test)) |
| He et al. 2022 (18) | MI [30]; healthy controls [30] | plasma sEV | ELISA | LDLR | lower in MI (p<0.001 (t-test)) |
|  |  |  |  | APOA5 | lower in MI (p<0.001 (t-test)) |
| Li et al. 2021 (19) | ACS (uAP 160; MI 50); healthy controls [50] | plasma sEV | WB, ELISA | Cyr61 | elevated in ACS (AUC=0.763 95% CI=0.705-0.822 p<0.01) |
| Dekker et al. 2020 (20) | CCS [187]; healthy controls [257] | LDL plasma subfraction sEV | immuno-bead assay | CD14 | predictor of stress-induced ischemia (OR 1.54, 95% CI=1.09-2.16 p=0.014) |
|  |  |  |  | Cystatin C | predictor of stress-induced ischemia (OR 1.70, 95% CI=1.17-2.46 p=0.006) |
|  |  | HDL plasma subfraction sEV |  | CD14 | predictor of stress-induced ischemia (OR 1.46, 95% CI=1.02-2.11 p=0.042) |
|  |  |  |  | Cystatin C | predictor of stress-induced ischemia (OR 1.35, 95% CI=1.04-1.76 p=0.026) |
|  |  |  |  | Serpin C1 | predictor of stress-induced ischemia (OR 1.31, 95% CI=1.04-1.66 p=0.024) |
|  |  |  |  | Serpin G1 | predictor of stress-induced ischemia (OR 1.37, 95% CI=1.04-1.80 p=0.027) |
| Dekker et al. 2020 (21) | uAP [83]; healthy controls [186] | TEX plasma subfraction sEV | ECLIA | Cystatin C | decreased in uAP (OR 0.93 95% CI=0.88-0.99) |
| Liu et al. 2023 (29) | validation cohort: MI [62]; healthy controls [31] | plasma sEV | miRNA-seq; validation: RT-qPCR | miR-4516 | elevated in MI (AUC=0.9809 p<0.0001) |
|  |  |  |  | miR-203 | elevated in MI (AUC=0.6823 p=0.006) |
|  |  |  | ELISA | SFRP1 | elevated in MI (AUC=0.9603 p<0.0001) |
| Li et al. 2021 (30) | ACS (STEMI, NSTEMI, uAP 21; 23; 19); healthy controls [25] | plasma sEV | RT-qPCR | miR-146a | elevated in ACS STEMI (AUC=0.8305 95% CI=0.6853-0.9756 p=0.0001) NSTEMI (AUC=0.8696 95% CI=0.7659-0.9732 p<0.0001) uAP (AUC=0.7916 95% CI= 0.6506-0.9326 p=0.001) |
| Wang et al. 2021  (31) | validation cohort: MI [12]; healthy controls [9] | plasma sEV | miRNA-seq; validation: RT-qPCR | miR-342-3p | decreased in MI (p<0.05 (t-test)) |
| Su et al. 2020 (32) | validation cohort: MI [30]; CCS [30] | plasma sEV | microarray; validation: RT-qPCR | miR-4507 | decreased in MI (AUC=0.772 95% CI=0.549-0.820 p=0.02) |
|  |  |  |  | miR-1915-3p | decreased in MI (AUC=0.684 95% CI=0.550-0.890 p<0.01) |
|  |  |  |  | miR-3656 | decreased in MI (AUC=0.771 95% CI=0.648-0.895 p<0.01) |
| Zhao et al. 2019 (33) | validation cohort: MI [14]; stable AP [10]; healthy controls [14] | plasma sEV | microarray; validation: RT-qPCR | miR-183 | decreased in controls, compared to diseased patients (p<0.05 (t-test)); elevated in MI compared to stable AP (p<0.01 (t-test)) |
| Han et al. 2023 (34) | validation cohort: CCS [36]; healthy controls [36] | plasma sEV | miRNA-seq; validation: RT-qPCR | let-7c-5p | decreased in CCS (AUC=0.8472 95% CI=0.7534-0.9411 p<0.0001) |
|  |  |  |  | miR-335-3p | elevated in CCS (AUC=0.8029 95% CI=0.7014-0.9043 p<0.0001) |
|  |  |  |  | miR-652-3p | decreased in CCS (AUC=0.8009 95% CI=0.7005-0.9013 p<0.0001) |
| Zhang et al. 2020 (35) | CCS [20]; healthy controls [20] | serum sEV | RT-qPCR | miR-942-5p | elevated in CCS (AUC=0.693 95% CI=0.527-0.858 p=0.037) |
|  |  |  |  | miR-149-5p | elevated in CCS (AUC=0.702 95% CI=0.536-0.869 p=0.028) |
|  |  |  |  | miR-32-5p | elevated in CCS (AUC=0.691 95% CI=0.525-0.858 p=0.039) |
| He et al. 2021 (36) | MI [10]; CAD [8]; healthy controls [10] | plasma sEV | RNA sequencing | ALPL | elevated in MI (AUC=0.99 95% CI=0.9484-1.000 p=0.0002) |
|  |  |  |  | CXCR2 | elevated in MI (AUC=0.98 95% CI=0.8478-1.000 p=0.0005) |
| Wang et al. 2023 (37) | STEMI [64], healthy controls [64] | plasma sEV | microarray and miRNA-seq datasets screened; validation: RT-qPCR | exo-circ-0020887 | elevated in STEMI (AUC=0.85 p<0.001) |
|  |  |  |  | exo-circ-0009590 | elevated in STEMI (AUC=0.80 p<0.001) |
| Chen et al. 2020 (38) | STEMI [47]; uAP [24], healthy controls [27] | serum sEV | RT-qPCR | NEAT1 | elevated in STEMI compared to uAP and controls (AUC=0.822 95% CI= 0.740-0.903 p<0.01) |
|  |  |  | immuno-blotting | MMP-9 | elevated in STEMI (AUC=0.793 95% CI=0.702-0.883 p<0.01) |
|  |  |  | RT-qPCR | miR-204 | decreased in STEMI compared to uAP and controls (AUC=0.743 95% CI= 0.646-0.840 p<0.01) |
| Zheng et al. 2020 (39) | 2. validation cohort: MI [85]; healthy controls [48] | plasma sEV | RNA-seq; validation: RT-qPCR | ENST00000556899.1 | elevated in MI (AUC=0.661 95% CI=0.560-0.762 p<0.002) |
|  |  |  |  | ENST00000575985.1 | elevated in MI (AUC=0.750 95% CI=0.661-0.838 p<0.001) |
| Zheng et al. 2023 (40) | 2. validation cohort: CCS [100]; controls [48] | plasma sEV | RNA-seq; validation: RT-qPCR | ENST00000424615.2 | elevated in CCS (AUC=0.654 95% CI=0.562-0.746 p=0.002) |
|  |  |  |  | ENST00000560769.1 | elevated in CCS (AUC=0.722 95% CI=0.627-0.817 p<0.001) |
| Liu et al. 2023 (41) | 2. validation cohort: CCS [100]; controls [48] | plasma sEV | RNA-seq; validation: RT-qPCR | exo-hsa_circ_0075269 | elevated in CCS (AUC=0.761 95% CI=0.669-0.852 p<0.001) |
|  |  |  |  | exo-hsa_circ_0000284 | elevated in CCS (AUC=0.623 95% CI=0.522-0.724 p=0.015) |
| Wei et al. 2020 (46) | validation cohort: AF [20]; SR controls [20] | plasma sEV | miRNA-sequencing; validation: RT-qPCR | miR-92b-3p | elevated in AF (p<0.005 (t-test)) |
|  |  |  |  | miR-1306-5p | elevated in AF (p<0.005 (t-test)) |
|  |  |  |  | miR-let-7b-3p | elevated in AF (p<0.005 (t-test)) |
| Mun et al. 2019 (47) | validation cohort: persistent AF [40]; SVT controls [20] | plasma sEV | microarray; validation: RT-qPCR | miR-107 | elevated in AF (p<0.001 (t-test)) |
|  |  |  |  | miR-320d | elevated in AF (p<0.001 (t-test)) |
|  |  |  |  | miR-103a-3p | elevated in AF (p<0.01 (t-test)) |
|  |  |  |  | miR-486-5p | elevated in AF (p<0.01 (t-test)) |
|  |  |  |  | let-7b-5p | elevated in AF (p<0.001 (t-test)) |
| Wang et al. 2019 (48) | validation cohort: AF [40]; SR controls [20] | plasma sEV | miRNA-seq; validation: RT-qPCR | miR-483-5p | elevated in AF (p<0.001 (t-test)) |
|  |  |  |  | miR-142-5p | decreased in AF (p=0.026 (t-test)) |
|  |  |  |  | miR-223-3p | decreased in AF (p=0.011 (t-test)) |
|  |  |  |  | miR-223-5p | decreased in AF (p=0.028 (t-test)) |
| Chen et al. 2021 (50) | AF [20]; healthy controls [20] | serum sEV | RT-qPCR | MIAT | elevated in AF (p<0.05 (t-test)) |
| Zhang et al. 2023 (52) | validation cohort: DCM with HF [10]; healthy controls [6] | plasma sEV | miRNA-seq; validation: RT-qPCR | miR-6511b-3p | elevated in DCM (p<0.05 (Wilcoxon-test)) |
|  |  |  |  | miR-6741-5p | elevated in DCM (p<0.05 (Wilcoxon-test)) |
|  |  |  |  | miR-423-5p | elevated in DCM (p<0.05 (Wilcoxon-test)) |
|  |  |  |  | miR-3138 | elevated in DCM (p<0.05 (Wilcoxon-test)) |
|  |  |  |  | miR-1304-3p | elevated in DCM (p<0.05 (Wilcoxon-test)) |
|  |  |  |  | miR-5010-5p | elevated in DCM (p<0.05 (Wilcoxon-test)) |
|  |  |  |  | miR-1306-3p | elevated in DCM (p<0.05 (Wilcoxon-test)) |
| Wang et al. 2021 (53) | CHF [10]; healthy controls [5] | serum sEV | GEO and HMDD datasets screened; validation: RT-qPCR | miR-320a | elevated in CHF (p<0.05 (t-test)) |
|  |  |  | ELISA | sST2 | elevated in CHF (p<0.01 (t-test)) |
| Verbree-Willemsen et al. 2020 (54) | HF [141]; controls without HF [222] | HDL plasma subfraction EV | bead-based immuno-assay | Cystatin C | elevated in HF (OR=2.43 97.5% CI=1.85-3.27 p<0.001) |
|  |  |  |  | CD14 | elevated in HF (OR=1.94 97.5% CI=1.49-2.56 p<0.001) |
|  |  | LDL plasma subfraction EV |  | Cystatin C | elevated in HF (OR=2.02 97.5% CI=1.55-2.68 p<0.001) |
|  |  |  |  | Serpin G1 | decreased in HF (OR=0.57 97.5% CI=0.43-0.74 p<0.001) |
|  |  |  |  | Serpin F2 | decreased in HF (OR=0.71 97.5% CI=0.56-0.9 p<0.001) |
|  |  | TEX plasma subfraction EV |  | Cystatin C | elevated in HF (OR=2.09 97.5% CI=1.61-2.75 p<0.001) |
|  |  |  |  | CD14 | elevated in HF (OR=1.48 97.5% CI=1.17-1.90 p<0.001) |
|  |  |  |  | SerpinG1 | elevated in HF (OR=1.34 97.5% CI=1.06-1.71 p<0.001) |
| Wu et al. 2018 (55) | DCM-AHF [43]; healthy controls [34] | serum sEV | RT-qPCR | miR-92b-5p | elevated in DCM-AHF (AUC=0.808 95% CI=0.712-0.903 p<0.001) |
| Wu et al. 2018 (56) | HFrEF-AHF [28]; healthy controls [30] | serum sEV | RT-qPCR | miR-92b-5p | elevated in HFrEF-AHF (AUC=0.844 p<0.001) |
| Roura et al. 2018 (57) | DCM [20]; healthy controls [15] | plasma EV | LC-MS/MS | Fibrinogen | clustered in EV derived from DCM patients (p<0.05 (t-test)) |
|  |  |  |  | Serotransfer-rin |  |
|  |  |  |  | alfa-1-antitrypsin |  |
|  |  |  |  | apolipoprotein family members (C-I, C-III, D, H, beta-2-glycoprotein, J) |  |
| Zhang et al. 2023 (58) | validation cohort: FM [15]; healthy controls [15] | plasma sEV | microarray; validation: RT-qPCR | miR-146a-5p | elevated in FM (AUC=0.987 95% CI=0.9552-1.000) |
|  |  |  |  | miR-23a-3p | elevated in FM (AUC=0.9333 95% CI=0.8272-1.000) |
|  |  |  |  | miR-27a-3p | elevated in FM (AUC=0.9244 95% CI=0.8133-1.000) |
| Zhang et al. 2021 (61) | validation cohort: FM [60]; healthy controls [60] | serum sEV | miRNA-seq; validation: RT-qPCR | miRNA panel (miR-155 and miR-320a) | elevated in FM (AUC=0.944 95% CI=0.912-0.976) |
| Celik et al. 2023 (65) | validation cohort: ACR >2R [16]; non-rejecting HTX controls [33] | plasma EV | proximity extension assay; validation: nanoscale flow cytometric assay | TWEAK^+^ EV number | elevated in ACR (p=0.042 (t-test) AUC=0.582 95% CI=0.41-0.76 p=0.358) |
| Castellani et al. 2020 (66) | validation cohort: ACR [11]; AMR [9]; non-rejecting controls [R0] [33] | plasma EV | multiplex flow cytometry | CD3 | elevated in ACR compared to R0 (AUC=0.848 95% CI=0.736-0.961 p<0.001) |
|  |  |  |  | CD2 | elevated in ACR compared to R0 (AUC=0.829 95% CI=0.704-0.955 p<0.001) |
|  |  |  |  | ROR1 | elevated in ACR compared to R0 (AUC=0.771 95% CI=0.627-0.915 p<0.008) elevated in AMR compared to R0 (AUC=0.879 95% CI=0.776-0.981 p<0.001) |
|  |  |  |  | SSEA-4 | elevated in ACR compared to R0 (AUC=0.832 95% CI=0.711-0.952 p<0.001) elevated in AMR compared to R0 (AUC=0.820 95% CI=0.692-0.947 p<0.004) |
|  |  |  |  | HLA-I | elevated in ACR compared to R0 (AUC=0.939 95% CI=0.869-1.000 p<0.001) elevated in AMR compared to R0 (AUC=0.872 95% CI=0.757-0.988 p<0.001) |
|  |  |  |  | CD41b | elevated in ACR compared to R0 (AUC=0.815 95% CI=0.653-0.978 p<0.002) elevated in AMR compared to R0 (AUC=0.778 95% CI= 0.619-0.937 p<0.011) |
|  |  |  |  | CD19 | elevated in AMR compared to R0 (AUC=0.795 95% CI=0.648-0.942 p<0.007) |
|  |  |  |  | HLA-II | elevated in AMR compared to R0 (AUC=0.788 95% CI=0.653-0.922 p<0.009) |
|  |  |  |  | CD25 | elevated in AMR compared to R0 (AUC=0.727 95% CI=0.568-0.886 p<0.039) |
|  |  |  |  | CD326 | elevated in AMR compared to R0 (AUC=0.788 95% CI=0.597-0.979 p<0.009) |
|  |  |  |  | CD20 | elevated in AMR compared to R0 (AUC=0.798 95% CI=0.651-0.945 p<0.007) |

**Table 3** Overview of selected publications about the prognostic potential of EV in CVD.

EV: extracellular vesicle, AUC: area under curve, STEMI: ST-elevation myocardial infarction, VA-ECMO: veno-arterial extracorporeal membrane oxygenation, LVEF: left ventricular ejection fraction, NTA: nanoparticle tracking analysis, ELISA: enzyme-linked immunosorbent assay, MVO: microvascular obstruction, MSI: myocardial salvage index, CAD: coronary artery disease, CCS: chronic coronary syndrome, RT-qPCR: real-time quantitative PCR, miRNA-seq: micro-RNA sequencing, MACE: major adverse cardiovascular event, HR: hazard ratio, CI: confidence interval, CEA: carotid endarterectomy, LDL: low-density lipoprotein, HDL: high-density lipoprotein, TEX: tiny extracellular fraction, miR: micro-RNA, LC-MS/MS: liquid chromatography tandem mass spectrometry, Cer: ceramide, PC: phosphatidylcholine, TNFRSF14: tumor necrosis factor receptor superfamily member 14, MMP9: matrix-metalloproteinase 9, TFF3: trefoil factor 3, SELP: P-selectin, GRN: granulins, LTBR: lymphotoxin-beta receptor, GDF15: growth/differentitation factor 15, DLK1: protein delta homolog 1, PCSK9: proprotein convertase subtilisin/kexin type 9, U-PAR: urokinase plasminogen activator surface receptor, OPN: osteopontin, JAM-A: junctional adhesion molecule A, Gal4: galectin-4, SHPS-1: tyrosine-protein phosphatase non-receptor type substrate 1, CCL15: C-C motif chemokine 15, IGFBP-7: insulin-like growth factor-binding protein 7, IL18BP: interleukin-18-binding protein, RARRES2: retinoic acid receptor responder protein 2, TNF-R1: tumor necrosis factor receptor 1, IGFBP-2: insulin-like growth factor-binding protein 2, CCL16: C-C motif chemokine 16, OR: odds ratio, FH: familial hypercholesterolemia, AF: atrial fibrillation, ddPCR: droplet digital PCR, CKD: chronic kidney disease, vWF: von Willebrand factor, LV: left ventricle, LVR: left ventricular remodeling, MI: myocardial infarction, HF: heart failure

| Reference | Groups [N] | EV subpopulation | Detection method | EV-bound target or variable | Endpoint/Risk associated with | Study finding (statistical significance)) |
| --- | --- | --- | --- | --- | --- | --- |
| Siegel et al. 2021 (69) | STEMI [19]; patients on VA-ECMO [18] | plasma EV | flow cytometry | Annexin V^+^ EV number | patient mortality | increased in non-survivors of VA-ECMO (AUC=0.79 p=0.05 (logistic regression analysis)) |
|  |  |  |  | caveolin-3^+^ EV number | moderately-severely reduced left ventricular function | increased in patients with reduced LVEF (p=0.03 (t-test)) |
| Zara et al. 2023 (13) | STEMI [42] | plasma sEV | NTA | sEV size | MVO/MSI | smaller EV size is associated with MVO and lower MSI (OR=0.93 95% CI=0.87-0.98) |
|  |  |  | ELISA | platelet marker CD41-CD61 expression |  | lower expression is associated with MVO (OR=0.04 95% CI=0-0.61) |
| Han et al. 2023 (70) | validation cohort: CAD and hyperglycemia [75]; CAD and normoglycemia [75] | serum sEV | microarray; validation: RT-qPCR | hsa-let-7b-5p | SYNTAX score | upregulated in hyperglycemia (p<0.001 (Spearman correlation analysis)) |
| Zheng et al. 2023 (40) | 2. validation cohort: CCS [100]; controls [48] | plasma sEV | miRNA-seq; validation: RT-qPCR | ENST00000560769.1 | CAD severity | higher levels associated with disease severity (p=0.028 (Spearman correlation)) |
| Jansen et al. 2014 (82) | CCS [181] | plasma EV | RT-qPCR | miR-126 | MACE-free 6-year survival rate | higher levels were associated with higher survival rates (HR=0.485 95% CI=0.278-0.846 p=0.011) |
|  |  |  |  | miR-199a |  | higher levels were associated with higher survival rates (HR=0.458 95% CI=0.222-0.945 p=0.035) |
| Wang et al. 2023 (37) | STEMI [64] | plasma sEV | microarray and RNA-seq datasets screened; validation: RT-qPCR | circ-0020887 | MACE-free 1-year survival rate | higher levels are associated with lower survival (p=0.009 (Kaplan-Meier analysis)) |
|  |  |  |  | circ-0009590 |  | higher levels are associated with lower survival (p<0.001 (Kaplan-Meier analysis)) |
| Timmerman et al. 2021 (71) | 864 CEA [from which 137 MACE] | HDL plasma subfraction sEV | electro-chemolu-mines-cence immuno-assay | CD14 | 3-year postoperative risk of MACE | higher levels are associated with increased risk of MACE (HR=1.30 95% CI=1.15-1.48 p<0.001) |
|  |  |  |  | Cystatin C |  | higher levels are associated with increased risk of MACE (HR=1.22 95% CI=1.06-1.42 p=0.007) |
|  |  |  |  | Serpin F2 |  | higher levels are associated with increased risk of MACE (HR=1.36 95% CI=1.16-1.61 p<0.001) |
|  |  |  |  | Serpin C1 |  | higher levels are associated with increased risk of MACE (HR=1.29 95% CI=1.10-1.51 p=0.002) |
| Timmerman et al. 2022 (83) | 873 CEA [from which 138 MACE] | LDL plasma subfraction EV | LC-MS/MS | Cer(d18:1/24:1) | 3-year postoperative risk of MACE | higher levels are associated with increased risk of MACE (HR=1.24 95% CI=1.01-1.53 p=0.04) |
|  |  |  |  | Cer(d18:1/16:0)/PC(16:0/22:5) ratio |  | higher ratio is associated with increased risk of MACE (HR=1.26 95% CI=1.04-1.52 p=0.016) |
|  |  | TEX plasma subfraction EV |  | Cer(d18:1/16:0)/Cer(d18:1/24:0) ratio |  | higher ratio is associated with increased risk of MACE (HR=1.34 95% CI= 1.06-1.70 p=0.016) |
|  |  |  |  | Cer(d18:1/18:0)/Cer(d18:1/24:0) ratio |  | higher ratio is associated with increased risk of MACE (HR=1.24 95% CI=1.01-1.51 p=0.042) |
|  |  |  |  | Cer(d18:1/24:1)/Cer(d18:1/24:0) ratio |  | higher ratio is associated with increased risk of MACE (HR=1.31 95% CI=1.08-1.58 p=0.005) |
| Verwer et al. 2023 (84) | CEA [88] | LDL plasma subfraction EV | proximity extension assay | TNFRSF14 | 3-year postoperative risk of MACE | higher levels are associated with increased risk of MACE (p=0.013 (Mann-Whitney U test)) |
|  |  |  |  | MMP9 |  | higher levels are associated with increased risk of MACE (p=0.036 (Mann-Whitney U test)) |
|  |  |  |  | TFF3 |  | higher levels are associated with increased risk of MACE (p=0.029 (Mann-Whitney U test)) |
|  |  |  |  | SELP |  | higher levels are associated with increased risk of MACE (p=0.032 (Mann-Whitney U test)) |
|  |  |  |  | GRN |  | higher levels are associated with increased risk of MACE (p=0.049 (Mann-Whitney U test)) |
|  |  |  |  | LTBR |  | higher levels are associated with increased risk of MACE (p=0.005 (Mann-Whitney U test)) |
|  |  |  |  | GDF15 |  | higher levels are associated with increased risk of MACE (p=0.011 (Mann-Whitney U test)) |
|  |  |  |  | DLK1 |  | higher levels are associated with increased risk of MACE (p=0.044 (Mann-Whitney U test)) |
|  |  |  |  | PCSK9 |  | higher levels are associated with increased risk of MACE (p=0.022 (Mann-Whitney U test)) |
|  |  |  |  | UPAR |  | higher levels are associated with increased risk of MACE (p=0.010 (Mann-Whitney U test)) |
|  |  |  |  | OPN |  | higher levels are associated with increased risk of MACE (p=0.004 (Mann-Whitney U test)) |
|  |  |  |  | JAMA |  | higher levels are associated with increased risk of MACE (p=0.024 (Mann-Whitney U test)) |
|  |  |  |  | Gal4 |  | higher levels are associated with increased risk of MACE (p=0.006 (Mann-Whitney U test)) |
|  |  |  |  | SHPS1 |  | higher levels are associated with increased risk of MACE (p=0.011 (Mann-Whitney U test)) |
|  |  |  |  | CCL15 |  | higher levels are associated with increased risk of MACE (p=0.036 (Mann-Whitney U test)) |
|  |  |  |  | IGFBP7 |  | higher levels are associated with increased risk of MACE (p=0.042 (Mann-Whitney U test)) |
|  |  |  |  | IL18BP |  | higher levels are associated with increased risk of MACE (p=0.033 (Mann-Whitney U test)) |
|  |  |  |  | RARRES2 |  | higher levels are associated with increased risk of MACE (p=0.036 (Mann-Whitney U test)) |
|  |  |  |  | TNFR1 |  | higher levels are associated with increased risk of MACE (p=0.011 (Mann-Whitney U test)) |
|  |  |  |  | IGFBP2 |  | higher levels are associated with increased risk of MACE (p=0.049 (Mann-Whitney U test)) |
|  |  |  |  | CCL16 |  | higher levels are associated with increased risk of MACE (p=0.047 (Mann-Whitney U test)) |
| Oggero et al. 2022 (85) | hypertensive [40] from which 20 had MACE | plasma EV | Imaging Flow Cytometry | CD14^+^ EV number | 3,5-year MACE-free survival | higher levels are associated with increased risk of MACE (p=0.09 (paired t-test); OR=3.74 95% CI=1.12-12.49 p=0.032) |
|  |  |  |  | CD14^+^/CD41^+^ EV number |  | higher levels are associated with increased risk of MACE (p=0.0012 (paired t-test); OR=1.39 95% CI=0.93-2.06 p=0.104) |
| Suades et al. 2019 (86) | FH patients developing MACE [92], not developing MACE [48] | plasma MV | flow cytometry | total number of AnxV^+^ MV | 3.3±2.6 years MACE-free survival | increased in patients developing MACE (p<0.05 Mann-Whitney U test) |
|  |  |  |  | CD45^+^ MV number |  | increased in patients developing MACE (p<0.01 Mann-Whitney U test) |
|  |  |  |  | CD15^+^ MV number |  | increased in patients developing MACE (p<0.05 Mann-Whitney U test) |
|  |  |  |  | CD14^+^/11b^+^ MV number |  | lower in patients developing MACE (p<0.05 Mann-Whitney U test) |
|  |  |  |  | PAC1^+^ MV number |  | increased in patients developing MACE (p<0.05 Mann-Whitney U test) |
|  |  |  |  | CD62P^+^ MV number |  | increased in patients developing MACE (p<0.01 Mann-Whitney U test) |
|  |  |  |  | TSP1^+^ MV number |  | increased in patients developing MACE (p<0.01 Mann-Whitney U test) |
|  |  |  |  | CD45^+^, CD15^+^, CD31^+^ CD41a^+^/61^+^, CD62P^+^ number combined |  | increased in patients developing MACE AUC: 0.745±0.043 (P<0.001; 95% CI, 0.661–0.830) |
| Escate et al. (87) | validation cohort: FH patients with MACE [72], FH patients without MACE [47], non-FH controls [30] | plasma EV | TaqMan Array; validation: RT-qPCR | miR-133a | 8 years MACE free survival | increased in patients developing MACE AUC=0.76±0.054 95% CI=0.66-0.87 p<0.001 HR=3.89 95%CI=1.88-8.07, p<0.001 |
| Mork et al. 2019 (88) | AF [13]; non-AF control [12] | plasma EV | Protein microarray platform | TF bearing EV number | potentially increased thrombogenicity in AF | higher levels in AF (p<0.05 (Mann-Whitney U test)) |
| Siwaponanan et al. 2022 (89) | validation cohort: AF [30]; cardiac healthy controls non-AF [30] | plasma EV | microarray; validation: ddPCR | miR-378-3p | risk of AF | increased in AF (OR=3.09 CI=1.24-7.68 p=0.015) |
|  |  |  |  | miR-339-3p |  | increased in AF (OR=2.04 CI=1.06-3.92 p=0.032) |
|  |  |  |  | miR-106b-3p |  | increased in AF (OR=2.58 CI=1.24-5.35 p=0.011) |
|  |  |  |  | miR-328-3p |  | increased in AF (OR=2.68 CI=1.07-6.67 p=0.035) |
|  |  |  |  | miR-535-3p |  | increased in AF (OR=2.75 CI=1.15-6.55 p=0.022) |
|  |  |  |  | miR-590-5p |  | increased in AF (OR=2.96 CI=1.24-7.04 p=0.014) |
| Lau et al. 2017 (90) | AF [160] | plasma EV | flow cytometry | CD31^+^ EV number (endothelial/platelet derived) | progressive worsening of renal function (CKD stage) | increasing levels in worsening of CKD (p<0.001 Spearman's correlation) |
| Lim et al. 2022 (95) | MI [198] from which adverse [96] and reverse [100] remodeling | LDL plasma subfraction EV | beads-based immunoassay | vWF:plasminogen ratio | reverse LV remodeling 6 months after MI | higher in reverse remodeling (AUC=0.674 95% CI=0.599-0.748 p<0.001) |
|  |  |  |  | SerpinC1:plas-minogen ratio |  | higher in reverse remodeling (AUC=0.712 95% CI=0.639-0.786 p<0.001) |
| Gasecka et al. 2021 (96) | MI [55] from which LV remodeling [12] and no remodeling [43] | plasma EV | flow cytometry | CD146^+^ EV number | adverse LV remodeling 6 months after MI | decreased in adverse LVR (AUC=0.77 95% CI=0.60-0.95 p=0.009) |
|  |  |  |  | CD235a^+^ EV number |  | decreased in adverse LVR (AUC=0.75 95% CI=0.57-0.92 p=0.018) |
|  |  |  |  | CD61^+^ EV number |  | decreased in adverse LVR (AUC=0.76 95% CI=0.58-0.94 p=0.014) |
|  |  |  |  | combined |  | (AUC=0.87 95% CI=0.73-1.00 p=0.0004) |
| Zheng et al. 2020 (39) | validation cohort: MI [85] | plasma sEV | RNA-seq; validation: RT-qPCR | ENST00000575985.1 | severe HF after MI | positively associated with risk (OR=1.046 95% CI=1.005-1.089 p=0.029) |
| Wang et al. 2018 (97) | HF [31]; healthy controls [31] | plasma sEV | RT-qPCR | miR-425 | HF potentially caused by fibrosis | downregulated in HF (p<0.05 (t-test)) |
|  |  |  |  | miR-744 |  | downregulated in HF (p<0.001 (t-test)) |
| Suades et al 2023 (101) | chronic HF [119], non-HF controls [21] | plasma MV | flow cytometry | PS^-^ EV number | having chronic HF | increased in cHF (p=0.007, Mann-Whitney U Test) |
|  |  |  |  | CD31^+^/PS^-^ EV number |  | increased in cHF (p=, Mann-Whitney U Test) |
|  |  |  |  | CD45^+^/PS^-^ EV number |  | increased in cHF (p=0.036, Mann-Whitney U Test) |
|  |  |  |  | CD31^+^/PS^-^ EV number |  | increased in cHF (p=0.002, Mann-Whitney U Test) |
|  |  |  |  | CD15^+^/PS^-^ EV number |  | increased in cHF (p=0.001, Mann-Whitney U Test) |
|  |  |  |  | CD56^+^/PS^-^ EV number |  | increased in cHF (p=0.005, Mann-Whitney U Test) |
|  |  |  |  | CX43^+^/PS^-^ EV number |  | increased in cHF (p=0.001, Mann-Whitney U Test) |
|  |  |  |  | CD31^+^/PS^+^ EV number |  | decreased in cHF (p<0.001, Mann-Whitney U Test) |
| Vilella-Figuerola et al. 2023 (102) | chronic HF [119], non-HF controls [21], ACS [58], non-ACS [24] | plasma EV | flow cytometry | PS^-^ EV number | having chronic HF | increased in cHF compared to ACS (p<0.001, Mann-Whitney U Test) |
|  |  |  |  | PS^+^ EV number |  | increased in ACS compared to cHF (p=0.02, Mann-Whitney U Test) |
|  |  |  |  | CD31^+^/PS^+^ EV number |  | increased in cHF compared to ACS (p<0.001, Mann-Whitney U Test) |
|  |  |  |  | CD31^+^/PS^+^ EV number |  | decreased in cHF compared to non-HF (p<0.001, Mann-Whitney U Test) |
|  |  |  |  | CD41^+^/PS^+^ EV number |  | decreased in cHF compared to non-HF (p<0.001, Mann-Whitney U Test) |
|  |  |  |  | CD31^+^ / CD41^+^ / PS^+^ EV number |  | decreased in cHF compared to non-HF (p<0.001, Mann-Whitney U Test) |
